# Integration of the PD-L1 inhibitor atezolizumab and WT1/DC vaccination into standard-of-care first-line treatment for patients with epithelioid malignant pleural mesothelioma—Protocol of the Immuno-MESODEC study

**DOI:** 10.1371/journal.pone.0307204

**Published:** 2024-07-15

**Authors:** Jolien Van den Bossche, Maxime De Laere, Koen Deschepper, Paul Germonpré, Yvan Valcke, Jan Lamont, Barbara Stein, Kirsten Van Camp, Charlotte Germonpré, Griet Nijs, Ella Roelant, Sébastien Anguille, Eva Lion, Zwi Berneman

**Affiliations:** 1 Center for Cell Therapy and Regenerative Medicine (CCRG), Antwerp University Hospital (UZA), Edegem, Belgium; 2 Laboratory of Experimental Hematology, Vaccine & Infectious Disease Institute (VAXINFECTIO), Faculty of Medicine and Health Sciences, University of Antwerp, Antwerp, Belgium; 3 Division of Pulmonary and Infectious Diseases, VITAZ, Sint-Niklaas, Belgium; 4 Respiratory Oncology & Integrated Cancer Cancer Ghent, AZ Maria Middelares, Ghent, Belgium; 5 Clinical Trial Center (CTC), CRC Antwerp, Antwerp University Hospital (UZA), University of Antwerp, Edegem, Belgium; 6 Division of Hematology, Antwerp University Hospital (UZA), Edegem, Belgium; Baylor College of Medicine, UNITED STATES OF AMERICA

## Abstract

Malignant pleural mesothelioma (MPM) is an aggressive cancer with a very poor prognosis. Recently, immune checkpoint inhibition (ICI) has taken center stage in the currently ongoing revolution that is changing standard-of-care treatment for several malignancies, including MPM. As multiple arguments and accumulating lines of evidence are in support of the existence of a therapeutic synergism between chemotherapy and immunotherapy, as well as between different classes of immunotherapeutics, we designed a multicenter, single-arm, phase I/II trial in which both programmed-death-ligand 1 (PD-L1) inhibition and dendritic cell (DC) vaccination are integrated in the first-line conventional platinum/pemetrexed-based treatment scheme for epithelioid MPM patients (Immuno-MESODEC, ClinicalTrials.gov identifier NCT05765084). Fifteen treatment-naïve patients with unresectable epithelioid subtype MPM will be treated with four 3-weekly (±3 days) chemo-immunotherapy cycles. Standard-of-care chemotherapy consisting of cisplatinum (75mg/m^2^) and pemetrexed (500mg/m^2^) will be supplemented with the anti-PD-L1 antibody atezolizumab (1200 mg) and autologous *Wilms’ tumor 1* mRNA-electroporated dendritic cell (WT1/DC) vaccination (8–10 x 10^6^ cells/vaccination). Additional atezolizumab (1680 mg) doses and/or WT1/DC vaccinations (8–10 x 10^6^ cells/vaccination) can be administered optionally following completion of the chemo-immunotherapy scheme. Follow-up of patients will last for up to 90 days after final atezolizumab administration and/or WT1/DC vaccination or 24 months after diagnosis, whichever occurs later. The trial’s primary endpoints are safety and feasibility, secondary endpoints are clinical efficacy and immunogenicity. This phase I/II trial will evaluate whether addition of atezolizumab and WT1/DC vaccination to frontline standard-of-care chemotherapy for the treatment of epithelioid MPM is feasible and safe. If so, this novel combination strategy should be further investigated as a promising advanced treatment option for this hard-to-treat cancer.

## Introduction

Malignant pleural mesothelioma (MPM) is a rare and highly aggressive cancer arising from the mesothelial surfaces of the pleural cavities. The occurrence of MPM is tightly associated with prior, mostly professional, asbestos exposure [1]. Although preventive measures to limit asbestos use and exposure have been around for several decades, the incidence of MPM is only expected to decrease very slowly for several years ahead due to the long latency between asbestos exposure and MPM development as well as the persistence of environmental exposure [2, 3]. Until recently, patients with advanced or unresectable disease had limited treatment options, with doublet chemotherapy consisting of a platinum compound, either cisplatin or carboplatin, and a folate antimetabolite, either pemetrexed or raltitrexed, being the only available first-line therapy, resulting in a dismal 5-year survival rate of less than 10% and a median overall survival (mOS) of 12.1–16.1 months [4–7].

Driven by the persisting unmet medical need for MPM, research efforts over the past decade have focused on novel treatment modalities to improve prognosis. This has led to some important breakthroughs, with immunotherapy, and especially immune checkpoint inhibitors (ICI), taking center stage in the revolution that changed standard-of-care treatment for these patients. In the CheckMate 743 trial, a durable survival benefit was observed in patients treated with ICI targeting the programmed cell death protein-1 (nivolumab, OPDIVO®) and the cytotoxic T-lymphocyte-associated protein-4 (ipilimumab, YERVOY®), as compared to platinum/pemetrexed-treated patients (mOS 18.1 months versus 14.1 months) [6, 8]. Based on these results, both the United States of America’s Food and Drug Administration and the European Medicines Agency approved this combinatorial ICI therapy as an alternative standard-of-care first-line treatment for unresectable MPM. It has to be noted that the beneficial effect of this dual ICI regimen was mainly driven by the subgroup of patients with non-epithelioid MPM, which is related to the inferior effect of chemotherapy in this patient population [9], underlining the continued relevance of chemotherapy in the treatment of epithelioid MPM.

Another immunotherapeutic approach that has elaborately been explored over the past decades as an adjuvant treatment modality for a range of malignancies, including MPM, is dendritic cell (DC) vaccination [10–12]. Being the most efficient professional antigen-presenting cells of the immune system, DC are potent activators of CD8+ T cells, which, as key effector cells of the adaptive immune system, are capable of recognizing and eradicating malignant cells in an antigen-specific manner. In this way, DC immunotherapy could contribute to the establishment of long-lasting antitumor immunity, thereby improving the patient’s outcome. Since the publication of the first clinical trial in 1996 [13], DC vaccination was repeatedly shown to be safe and well-tolerated, with side-effects generally being limited to local injection site reactions. Moreover, these studies as well as our own clinical data in patients with different hematological and solid malignancies, including MPM patients, have demonstrated that DC vaccination is capable of inducing immunological and clinical responses [11, 14–17].

Directly linked to the therapeutic potential of DC vaccination for MPM treatment, is the selection of a suitable antigenic target. Several tumor-associated antigens (TAAs) have been identified in malignant mesothelioma cells [18], of which the Wilms’ tumor antigen 1 (WT1) is of particular interest as it is overexpressed in up to 99% of all MPM cases [19] and thus serves as a potent biomarker in MPM diagnosis. In our first phase I/II trial for patients with solid tumors (NCT01291420), amongst which 10 MPM patients, autologous WT1-targeted DC (WT1/DC) vaccination administered as consolidation treatment following platinum/pemetrexed chemotherapy was shown to be feasible and safe. In addition, *in vivo* evidence of vaccine-elicited immunity was demonstrated in 9 out of 10 patients, resulting in a mOS of 35.7 months after a median follow-up of 55.9 months post-start chemotherapy [11], which compares favorably to the reported mOS of 16.7 months [8] and 22 months [20] for a similar cohort of chemotherapy-treated patients.

Based on these encouraging results, a subsequent phase I/II trial for MPM patients diagnosed with the epithelioid subtype was initiated to assess feasibility and safety of WT1/DC vaccination during and after first-line platinum/pemetrexed chemotherapy (MESODEC, NCT02649829). Historically, chemotherapy and immunotherapy were considered to be non-compatible treatment options, primarily due to the non-specific cytotoxic actions of the former. However, increased antigen exposure following chemotherapy-induced immunogenic tumor-cell damage, the preferential elimination of regulatory cells by certain chemotherapeutics and the enhanced immune responsiveness due to stimulatory cytokines produced in response to cytotoxic immune- and tumor-cell damage [21] actually support a stimulatory role for chemotherapy in DC-induced anti-cancer immunity, overthrowing this long-standing incompatibility paradigm. In addition, increased chemosensitivity after DC vaccination has also been reported in different types of cancer [22, 23], but the mechanisms behind this phenomenon remain elusive. At the second data analysis point (database lock on May 2, 2022), defined as the time when 20 participants had reached an event (death) or survived 22 months after diagnosis (i.e., date of tumor biopsy), 25 evaluable patients were enrolled. Safety evaluation showed occurrence of transient local mild adverse reactions (ARs) such as swelling, redness and itch at the injection site in the majority of patients (92.0%), while transient systemic mild ARs were reported in three patients (12.0%). No serious adverse events (SAEs) possibly, probably or definitely related to WT1/DC vaccination developed. At this interim analysis point, two-year OS was 65% (unpublished results), which compares favorably to the reported two-year OS of 33% for platinum/pemetrexed standard chemotherapy [6], indicating a potential clinical benefit for this patient population.

Overall, it is becoming increasingly clear that the direct and indirect immunological actions of conventional and next-generation therapeutics are crucial in determining anti-cancer treatment outcome. The design of optimized combinatorial treatment paradigms, which maximally exploit the synergistic interactions between different classes of therapeutics, could therefore further improve patient outcome. Besides combining chemo- and immunotherapy, combinatorial approaches including ICI and DC vaccination are also of particular interest, given their different modes of action on the immunological level, allowing for beneficial interactions to occur. Indeed, it is reasonable to assume that DC-induced immune responses can be hampered by an immunosuppressive tumor microenvironment, which can be overcome by ICI. ICI immunotherapies, on the other hand, typically require an ongoing T-cell response at the tumor location to be effective, which can be stimulated by DC vaccination. Improved anti-tumor responses suggestive of reciprocal stimulatory effects have indeed been observed when combining either CTLA-4- [24], PD-1- [25] or PD-L1- [25] targeted ICI with DC vaccination in murine models and in *in vitro* human models [26–28]. In our ongoing phase I/II trial (MESODEC, NCT02649829), 21 out of 25 patients were treated with ICI as second- or third-line treatment, while WT1/DC vaccination was continued (if residual WT1/DC vaccine aliquots were available). As the two-year survival rate of 65% observed at the interim data analysis point in our study (unpublished results) equally compares favorably to the reported two-year OS of 42% for the recently approved ICI combinatorial treatment regimen in first line [6], the potential occurrence of synergistic interactions between these two types of immunotherapies *in vivo* merits further investigation.

Based on this, we here present the protocol of a new phase I/II trial (Immuno-MESODEC) in which the anti-PD-L1 antibody atezolizumab (Tecentriq®) and WT1/DC vaccination are integrated in the standard-of-care chemotherapy schedule for MPM patients. Since the epithelioid subtype of MPM has been shown to have an increased baseline sensitivity to chemotherapy as compared to the non-epithelioid subtype and appears to benefit less explicitly from first-line ICI monotherapy [6], while being well responsive to WT1/DC vaccination and next-line ICI therapy, this trial is specifically designed for epithelioid MPM.

## Methods

### Study design and organization

The Immuno-MESODEC trial is an academic, multicenter, single-arm, phase I/II trial to investigate safety and feasibility of adding the anti-PD-L1 antibody atezolizumab and WT1/DC vaccination to first-line platinum/pemextrexed-based chemotherapy in epithelioid MPM patients. The Antwerp University Hospital (UZA, Edegem, Belgium) is both Sponsor and Coordinating Center. Two other Belgian hospitals, AZ Maria Middelares (AZMM, Ghent) and VITAZ (Sint-Niklaas) are responsible for patient recruitment and follow-up, treatment with standard-of-care chemotherapy, atezolizumab administrations, and treatment policy after disease progression. WT1/DC vaccines are produced at the GMP production facility anicells (Niel) and reconstituted and administered at the Antwerp University Hospital (UZA). The trial is conducted according to the principles of the Declaration of Helsinki and the ICH guideline for good clinical practice E6(R2) and has been approved by the Ethics Committee of the Antwerp University Hospital/University of Antwerp and the Belgian Federal Agency for Medicines and Health Products (FAMHP). The trial is registered in the EudraCT database with reference number 2021-003229-31. The ClinicalTrials.gov identifier is NCT05765084.

### Participants

The Immuno-MESODEC study aims to accrue 15 evaluable patients. Patients are considered evaluable if they have completed at least the first chemo-immunotherapy cycle. Inclusion and exclusion criteria are listed in Table 1. Informed consent must be given before any study-related procedure is performed.

**Table 1:** Inclusion and exclusion criteria.

**Inclusion criteria**
Age ≥ 18 years at the time of signing the informed consent form
Diagnosis with histologically proven, epithelioid and unresectable malignant pleural mesothelioma (MPM) (stage I-IV)
World Health Organization (WHO) performance status 0–1
Adequate hematologic and end-organ function, defined by the following laboratory test results, obtained around the time of screening: ◾ Absolute neutrophil count ≥ 1.5 x 10^9^/L (1500/μL) without granulocyte colony-stimulating factor support ◾ Lymphocyte count ≥ 0.5 x 10^9^/L (500/μL) ◾ Platelet count ≥ 100 x 10^9^/L (100,000/μL) without transfusion ◾ Hemoglobin ≥ 90 g/L (9 g/dL) Patients may be transfused to meet this criterion ◾ Aspartate aminotransferase (AST), alanine aminotransferase (ALT), and alkaline phosphatase (ALP) ≤ 2.5 x upper limit of normal (ULN), with the following exceptions: • Patients with documented liver metastases: AST and ALT ≤ 5 x ULN • Patients with documented liver or bone metastases: ALP ≤ 5 x ULN ◾ Total bilirubin ≤ 1.5 x ULN with the following exception: • Patients with known Gilbert disease: total bilirubin ≤ 3 x ULN ◾ Creatinine ≤ 1.5 x ULN ◾ Albumin ≥ 25 g/L (2.5 g/dL) ◾ For patients not receiving therapeutic anticoagulation: prothrombin international normalized ration (PT-INR) and activated partial thromboplastin time (aPTT) ≤ 1.5 x ULN
Negative human immunodeficiency virus (HIV) test at screening
Negative hepatitis B surface antigen (HBsAg) test at screening
Negative total hepatitis B core antibody (HBcAb) test at screening, or positive total HBcAb test followed by a negative hepatitis B virus (HBV) DNA test at screening ◾ The HBV DNA test will be performed only for patients who have a negative HBsAg test and a positive total HBcAb test.
Negative hepatitis C virus (HCV) antibody test at screening, or positive HCV antibody test followed by a negative HCV RNA test at screening. The HCV RNA test must be performed for patients who have a positive HCV antibody test.
Women of childbearing potential must have a negative serum or urine pregnancy test at the time of screening and agree to use effective contraception (<1% failure rate per year) before, during and for at least five months after the last atezolizumab administration or at least hundred days after the last WT1/DC vaccine administration (whichever takes longer). Men must agree to use effective contraception before, during and for at least hundred days after the last study treatment administration.
Willing and able to comply with the study protocol, as judged by the treating physician
**Exclusion criteria**
History of malignancy within 3 years prior to initiation of study treatment, with the exception of the cancer under investigation in this study and malignancies with a negligible risk of metastasis or death (e.g., 5-year overall survival (OS) rate > 90%), such as adequately treated carcinoma in situ of the cervix, non-melanoma skin carcinoma, localized prostate cancer, ductal carcinoma in situ, or stage I uterine cancer
Symptomatic, untreated, or actively progressing central nervous system (CNS) metastases. Asymptomatic patients with treated CNS lesions are eligible, provided that all of the following criteria are met: ◾ Measurable disease, per RECIST v1.1, must be present outside the CNS ◾ The patient has no history of intracranial hemorrhage or spinal cord hemorrhage ◾ The patient has not undergone stereotactic radiotherapy within 7 days prior to initiation of study treatment, whole-brain radiotherapy within 14 days prior to initiation of study treatment, or neurosurgical resection within 28 days prior to initiation of study treatment ◾ The patient has no ongoing requirement for corticosteroids as therapy for CNS disease ◾ If the patient is receiving anti-convulsant therapy, the dose is considered stable ◾ Metastases are limited to the cerebellum or the supratentorial region (i.e., no metastases to the midbrain, pons, medulla, or spinal cord) ◾ There is no evidence of interim progression between completion of CNS directed therapy and initiation of study treatment ◾ Asymptomatic patients with CNS metastases newly detected at screening are eligible for the study after receiving radiotherapy and/or surgery, with no need to repeat the screening brain scan
History of leptomeningeal disease
Active or history of autoimmune disease or immune deficiency, including, but not limited to, myasthenia gravis, myositis, autoimmune hepatitis, systemic lupus erythematosus, rheumatoid arthritis, inflammatory bowel disease, anti-phospholipid antibody syndrome, Wegener granulomatosis, Sjögren syndrome, Guillain-Barré syndrome, or multiple sclerosis, with the following exceptions: ◾ Patients with a history of autoimmune-related hypothyroidism who are on thyroid replacement hormone are eligible for the study ◾ Patients with controlled type 1 diabetes mellitus who are on an insulin regimen are eligible for the study ◾ Patients with eczema, psoriasis, lichen simplex chronicus, or vitiligo with dermatologic manifestations only (e.g., patients with psoriatic arthritis are excluded) are eligible for the study provided all of following conditions are met: • Rash must cover < 10% of body surface area • Disease is well controlled at baseline and requires only low-potency topical corticosteroids • No occurrence of acute exacerbations of the underlying condition requiring psoralen plus ultraviolet A radiation, methotrexate, retinoids, biologic agents, oral calcineurin inhibitors, or high potency or oral corticosteroids within the previous 12 months
History of idiopathic pulmonary fibrosis, organizing pneumonia (e.g., bronchiolitis obliterans), drug-induced pneumonitis, or idiopathic pneumonitis, or evidence of active pneumonitis on screening chest computed tomography (CT) scan. History of radiation pneumonitis in the radiation field (fibrosis) is permitted.
Significant cardiovascular disease (such as New York Heart Association Class II or greater cardiac disease, myocardial infarction, or cerebrovascular accident) within 3 months prior to initiation of study treatment, unstable arrhythmia, or unstable angina
Major surgical procedure, other than for diagnosis, within 4 weeks prior to initiation of study treatment, or anticipation of need for a major surgical procedure during the study
Severe infection within 4 weeks prior to initiation of study treatment, including, but not limited to, hospitalization for complications of infection, bacteremia, or severe pneumonia, or any active infection that could impact patient safety
Prior treatment for MPM
Treatment with therapeutic oral or IV antibiotics within 2 weeks prior to initiation of study treatment. Patients receiving prophylactic antibiotics (e.g., to prevent a urinary tract infection or chronic obstructive pulmonary disease (COPD) exacerbation) are eligible for the study.
Prior allogeneic stem cell or solid organ transplantation
Use of any investigational agent within 28 days before study enrollment
Pregnant or breastfeeding. Female subjects who are breastfeeding should discontinue nursing prior to the first dose of study treatment and until at least hundred days after the last study treatment administration.
Treatment with a live, attenuated vaccine within 4 weeks prior to initiation of study treatment, or anticipation of need for such a vaccine during atezolizumab treatment or within 5 months after the final dose of atezolizumab
Current treatment with anti-viral therapy for HBV
Prior treatment with CD137 agonists or immune checkpoint blockade therapies, including anti-CTLA-4, anti-PD-1, and anti-PD-L1 therapeutic antibodies
Treatment with systemic immunosuppressive medication (including, but not limited to, corticosteroids, cyclophosphamide, azathioprine, methotrexate, thalidomide, and anti-tumor necrosis factor-α [TNF-α] agents) within 2 weeks prior to initiation of study treatment, or anticipation of need for systemic immunosuppressive medication during study treatment, with the following exceptions: ◾ Patients who received acute, low-dose systemic immunosuppressant medication or a one-time pulse dose of systemic immunosuppressant medication (e.g., 48 hours of corticosteroids for a contrast allergy) may be eligible for the study after medical monitor confirmation has been obtained ◾ Patients who received mineralocorticoids (e.g., fludrocortisone), inhaled or low dose corticosteroids for COPD or asthma, or low-dose corticosteroids for orthostatic hypotension or adrenal insufficiency are eligible for the study.
Treatment with systemic immunostimulatory agents (including but not limited to interferons or interleukin-2) within 4 weeks or 5 drug-elimination half-lives of the drug, whichever is longer, prior to initiation of study treatment
History of severe allergic anaphylactic reactions to chimeric or humanized antibodies or fusion proteins
Known hypersensitivity to Chinese hamster ovary cell products or to any component of the atezolizumab formulation
Any other condition, either physical or psychological, or reasonable suspicion thereof on clinical or special investigation, which contraindicates the use of atezolizumab, pemetrexed, cisplatin/carboplatin and/or WT1/DC vaccination, or may negatively affect patient compliance, or may place the patient at higher risk of potential treatment complications

### Study objectives and outcome measurements

#### Primary objective and outcome measurements

The primary objective of this phase I/II trial is to investigate the feasibility and safety of adding both atezolizumab and WT1/DC vaccination to first-line platinum/pemetrexed-based chemotherapy. The measure for feasibility is the proportion of patients who successfully complete the study treatment schedule, where the denominator is the total number of patients enrolled in the study. Safety is evaluated based on the occurrence of (severe) adverse events ((S)AEs), including the proportion of evaluable patients who experienced AEs and/or SAEs that are possibly, probably or definitely related to pemetrexed and/or cisplatin/carboplatin and/or atezolizumab and/or WT1/DC vaccination. For reporting AEs, the National Cancer Institute’s Common Terminology Criteria for Adverse Events (CTCAE) and Common Toxicity Criteria (CTC) are used [29].

#### Secondary objectives and outcome measurements

The secondary objectives of the Immuno-MESODEC trial are to assess indicators of clinical activity and to determine immunogenicity of first-line platinum/pemetrexed-based chemotherapy when combined with atezolizumab administration and WT1/DC vaccination.

Patients are followed for clinical response, disease progression and survival. Tumor evolution assessment is performed according to the latest modified Response Evaluation Criteria In Solid Tumors (mRECIST) for malignant pleural mesothelioma and RECIST 1.1 criteria for metastatic lesions [30]. Best overall response (BOR), duration of response (DOR), disease control rate (DCR), objective response rate (ORR), progression free survival (PFS) and overall survival (OS) for first-line treatment are determined as outcome measurements for clinical efficacy. BOR is determined per patient as the best overall response designation. The BOR categories are complete response (CR), partial response (PR), stable disease (SD) and progressive disease (PD). DOR is calculated for patients with an objective response as the time between the first date of the first documented tumor response (CR or PR) and the subsequent date of the objectively documented PD or death, whichever occurs first, or the last available tumor assessment in case of censoring. DCR is defined as the proportion of patients whose BOR is either CR, PR or SD, where the denominator is the total number of evaluable patients. ORR is defined as the proportion of patients whose BOR is either CR or PR, where the denominator is the total number of evaluable patients. PFS is calculated as the time between start of first-line treatment and the date of PD or death due to any cause, whichever occurs first. At the time of analysis, patients without a recorded event are censored at the time of the last objective disease assessment. OS is calculated as the time between diagnosis or start of first-line treatment (as specified) and death due to any cause. Those still alive at the time of analysis are censored at the time they were last known to be alive.

To determine the immunogenicity of the proposed chemo-immunotherapy regimen, immunomonitoring studies are performed on blood samples collected at baseline and at defined time points throughout the study scheme. Outcome measures for immunogenicity include, but is not limited to, the occurrence of functional WT1-specific T cell responses.

#### Exploratory objectives and outcome measurements

The first exploratory objective of this phase I/II trial is to evaluate general quality-of-life (QoL) and disease-related symptoms over the treatment course, including association with clinical outcome, for which patients are asked to fill out EQ-5D-5L [31] and LCSS-Meso [32, 33] questionnaires at defined time points.

In the context of biomarker evaluation, which is another exploratory objective, tumor biopsies obtained at diagnosis (if available) are subjected to immunohistological assessments to characterize the tumor and the tumor microenvironment for attributes with known relevance to MPM pathology and immunotherapy response (e.g. WT1 expression, lymphocyte infiltration, PD-1/PD-L1 expression) in order to confirm their potential relationship to treatment response and patient outcome.

### Study procedures

**Fig 1:**
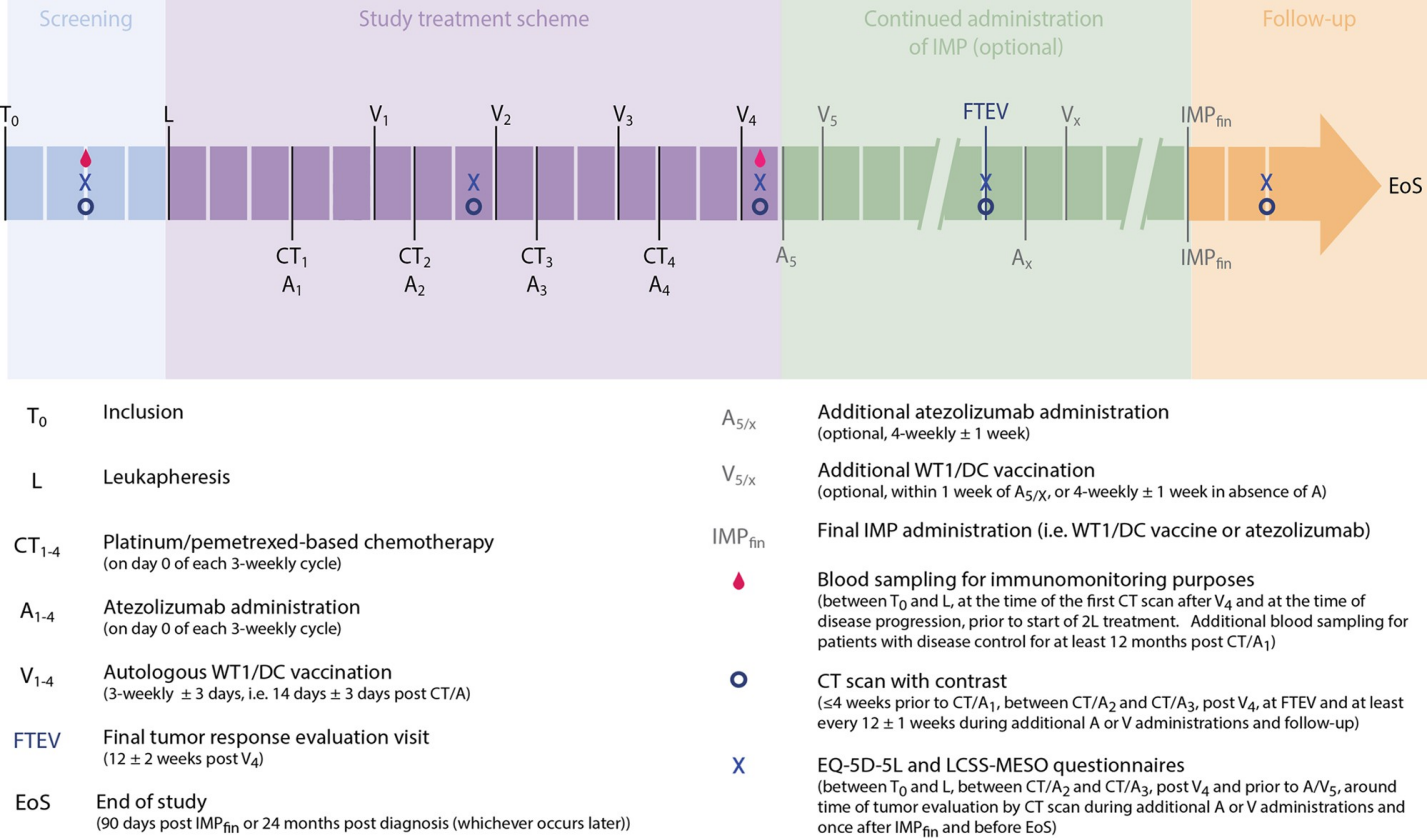
Schematic overview of the study-related procedures for participants of the Immuno-MESODEC trial.

An overview of the study related procedures and timelines is presented in Fig 1.

#### Consent and screening

After signing the informed consent form, patients are evaluated for eligibility to ensure that all inclusion and exclusion criteria are met. All screening evaluations should be performed within 4 weeks before leukapheresis. Confirmation of diagnosis and eligibility to undergo standard-of-care chemotherapy and atezolizumab administrations are performed by investigators located at AZMM and VITAZ. Eligibility to undergo leukapheresis and WT1/DC vaccination is verified by the responsible physicians at UZA’s Divisions of Nephrology and Hematology, under responsibility of UZA’s Cell and Tissue bank.

#### Leukapheresis and WT1/DC vaccine production

Clinical-grade WT1/DC vaccines are prepared as previously described [16, 17], starting from autologous leukapheresis material obtained by leukapheresis of the non-mobilized donor using the Spectra Optia device (Terumo, Leuven, Belgium). In brief, monocytes are isolated from the apheresis product by means of immunomagnetic bead selection using the CliniMACS® Cell Separation System (Miltenyi Biotec, Germany). Subsequently, isolated CD14+ monocytes are differentiated *ex vivo* into immature DCs in the presence of 800–1060 IU/mL granulocyte macrophage-colony stimulating factor and 250 IU/mL interleukin-4 for 6 days. On day 6, cells are matured through addition of 20 ng/mL tumor necrosis factor a, 2.5 μg/mL prostaglandin E2 and 10 μg/mL pyrogen-free keyhole limpet hemocyanin (KLH). Two days later, mature DCs are harvested and washed for downstream antigen loading through mRNA electroporation using the GenePulser Xcell electroporation device (BioRad, Ghent, Belgium). The WT1-DC-lysosomal associated membrane protein (LAMP) construct, with an incorporated Sig-DC-LAMP major histocompatibility complex (MHC) class 2-skewing signal and deletion of the WT1 nuclear localization signal [34], inserted in a plasmid vector, is used as a template to generate mRNA by *in vitro* transcription (eTheRNA immunotherapies, Niel, Belgium). Following electroporation, WT1/DCs are cryopreserved and remain under embargo from release until the quality control test results have become available and all release criteria have been met. Quality control testing performed on the cryopreserved WT1/DC aliquots consists of determining cell count and viability, testing sterility, determining endotoxin contents, performing flow cytometric analysis of DC morphology and phenotype (CD86, HLA-DR, CCR7, CD80, CD83, CD14) and the presence of T-lymphocyte impurities (CD3), immunohistochemistry for WT1 protein expression and analysis of functional migratory capacity.

#### Study treatment schedule

Patients receive four 3-weekly (± 3 days) platinum/pemetrexed-based chemotherapy cycles in combination with four atezolizumab administrations and four WT1/DC vaccinations at day 0 and day 14 (± 3 days) of each chemotherapy cycle, respectively.

Folic acid should be taken orally daily beginning at least one week before the first chemotherapy dose and should be continued until four weeks after the last chemotherapy cycle. Vitamin B12 (1000 μg) should be given intramuscularly one to three weeks before the first dose of chemotherapy and repeated every nine weeks until four weeks after the last chemotherapy cycle. In addition, methylprednisolone (32 mg orally or equivalent in whatever formulation is available locally) should be given the day before, the day of and the day after pemetrexed dosing to reduce the risk of severe skin rash.

On the first day of each chemotherapy cycle (day 0), atezolizumab (1200 mg) should be administered as an intravenous (IV) infusion over 60 minutes (± 15 minutes), followed by pemetrexed (500 mg/m^2^) and cisplatin (75 mg/m^2^) as IV infusions over 10 minutes and 2 hours, respectively. If the first atezolizumab infusion is well-tolerated, all subsequent infusions may be delivered over 30 minutes (± 10 minutes).

On day 14 (± 3 days) of each cycle, 8–10 x 10^6^ WT1/DC in 500 μL saline solution with 5% human albumin are injected intradermally at 5 sites (100 μL/site) in the ventromedial region of the upper arm, 5–10 cm from the axillary lymph nodes. Between cycles, injection sites alternate between left and right arms. In case of a delay in chemotherapy administration, atezolizumab administration and WT1/DC vaccination can be delayed accordingly.

Chemo-immunotherapy is continued for 4 cycles (i.e., the study treatment scheme) or until unacceptable toxicity develops, the participant decides to withdraw from therapy or the investigator decides to stop it.

#### Continuation of atezolizumab treatment and/or WT1/DC vaccination

After completion of the study treatment schedule, atezolizumab administration and/or WT1/DC vaccination can optionally be continued, provided that consent of the participant was obtained and that residual WT1/DC vaccine aliquots are available. Additional atezolizumab doses (1680 mg) should be administered as an IV infusion over 30–60 minutes on a 4-weekly basis (±1 week), followed by intradermal WT1/DC vaccination (8–10 x 10^6^ WT1/DC in 500 μL saline solution with 5% human albumin, administered intradermally) within one week, if applicable.

In case of a delay in atezolizumab administration, WT1/DC vaccination can be delayed accordingly. Upon prolonged delay of atezolizumab administration, WT1/DC vaccination can be restarted when the investigators judge that the participant’s clinical situation justifies additional administrations.

Atezolizumab can be administered until disease progression or until unacceptable toxicity occurs, the participant decides to withdraw from therapy, or the investigator decides to stop it. Because of the possibility of an initial increase in tumor burden caused by immune-cell infiltration (i.e., pseudoprogression) and since patients who progress after first-line treatment have limited treatment options, patients may be considered for treatment with atezolizumab beyond radiographic disease progression per RECIST v1.1, in the absence of unacceptable toxicity, at the discretion of the investigator and after appropriate discussion with the patient and after obtaining informed consent. Patients will be permitted to continue atezolizumab if (i) there is evidence of clinical benefit, as determined by the investigator following a review of available data, (ii) symptoms and signs (including laboratory values) indicating unequivocal progression of disease are absent, (iii) a decline in WHO performance status score that can be attributed to disease progression is absent and (iv) tumor progression at critical anatomical sites (e.g., leptomeningeal disease), that cannot be managed by protocol-allowed medical interventions, is absent. Patients who continue treatment beyond radiographic disease progression per RECIST v1.1 should be closely monitored clinically and with a follow-up scan after 6 weeks or sooner if symptomatic deterioration occurs. Treatment should be discontinued if clinical deterioration due to disease progression occurs at any time, or if persistent disease growth is confirmed in a follow-up scan. In addition, patients should be discontinued for unacceptable toxicity or for any other signs or symptoms of deterioration attributed to disease progression as determined by the investigator after an integrated assessment of radiographic data and clinical status. In case atezolizumab is discontinued, WT1/DC vaccines can be continued with administrations at 4-weekly intervals (±1 week).

WT1/DC vaccinations can continue as long as residual vaccine aliquots are available or until unacceptable toxicity develops, the participant decides to withdraw from therapy or the investigator decides to stop it. In case of exhaustion of the WT1/DC vaccines, atezolizumab administrations can be continued at 4-weekly intervals (±1 week).

Upon disease progression, next-line treatment can be started at the investigator’s discretion based on the availability of treatment options, including continuation of WT1/DC vaccination, and in agreement with the patient. Detailed information about all subsequent lines of therapy is collected beyond disease progression for all patients.

#### Patient evaluation and follow-up

Patients are monitored clinically and radiographically at regular predetermined time points from inclusion until 90 days after final atezolizumab administration or WT1/DC vaccination or 24 months after diagnosis, whichever occurs later, to capture safety and efficacy data.

Patients undergo clinical examination (i) during the screening period, (ii) at each chemotherapy/atezolizumab administration visit and WT1/DC vaccination visit during the study treatment schedule, (iii) at each additional atezolizumab treatment visit or each third WT1/DC vaccination visit (in case of WT1/DC vaccination alone), (iv) at final tumor response evaluation visit (i.e., 12 weeks (± 2 weeks) after the fourth WT1/DC vaccination) and (v) every 12 weeks (± 1 week) during follow-up. During these visits, a physical examination is performed, including evaluation of vital signs, determination of the WHO performance status and review of concurrent drug use and AEs. Peripheral blood analysis is done as a safety measurement at following time points: (i) during the screening period, (ii) at each chemotherapy and/or atezolizumab administration visit, (iii) at final tumor response evaluation visit (i.e., 12 weeks (± 2 weeks) after the fourth WT1/DC vaccination) and (vi) at follow-up visits at the investigators’ discretion. In case WT1/DC vaccination is continued without atezolizumab administration, peripheral blood analysis is performed at each third additional WT1/DC vaccination visit. If a next-line treatment with continued WT1/DC vaccination is started, patient evaluation and peripheral blood analysis is performed at the day of administration of the next-line treatment. Additional blood samples for immunomonitoring purposes are collected (i) prior to leukapheresis, (ii) at the time of the first tumor evaluation after completion of the study treatment scheme and (iii) at the time of disease progression, prior to the start of the second-line treatment (if applicable). Patients with sustained disease control (SD, PR or CR) for at least 12 months after the start of first-line chemo-immunotherapy will at that point undergo an additional blood sample collection for immunomonitoring purposes.

The patients’ disease status is monitored with a CT-scan with contrast of the chest and upper abdomen (i) ≤ 4 weeks before the start of the first chemo-immunotherapy cycle, (ii) between the second and the third chemo-immunotherapy cycle, (iii) after the fourth WT1/DC vaccine, (iv) within 12 weeks (± 2 weeks) after the fourth WT1/DC vaccination (i.e. final tumor response evaluation visit, FTEV), at least every 12 weeks (± 1 week) during additional atezolizumab administrations and/or WT1/DC vaccinations and during follow-up. In case a CT-scan with contrast is contra-indicated, a CT-scan without contrast can be performed instead. If atezolizumab treatment is continued beyond disease progression, an additional CT-scan is performed ≤ 6 weeks following radiological disease progression, or sooner if symptomatic deterioration occurs. An MRI scan at all tumor evaluation points may be used as an alternative.

In addition, patients are asked to fill out EQ-5D-5L [31] and LCSS-Meso [32, 33] questionnaires to evaluate general and disease-related QoL (i) during the screening period, (ii) around the time of tumor evaluation between the second and third chemotherapy cycle, (iii) after the study treatment schedule, but before additional atezolizumab administrations or WT1/DC vaccinations, (iv) around the time of tumor evaluation during additional atezolizumab administrations or WT1/DC vaccinations, (v) after the final atezolizumab administration or WT1/DC vaccination, but before end of study.

### Data collection and monitoring

The Principal Investigator or designated staff, as documented on the delegation log, completes the electronic case report forms (eCRFs) for all patients using the RedCAP software. Data monitoring is performed by monitors of UZA’s Clinical Trial Center (CTC) to check the completeness of patients’ records, to ensure that all aspects of the protocol are followed and to verify compliance with GCP guidelines. Site staff respond to queries providing clarifications and/or corrections of the discrepancies. Full details on data monitoring procedures are available in the Data Monitoring Plan.

### Statistical analysis

This phase I/II trial was specifically designed to evaluate safety and feasibility in a small group of epithelioid-type MPM patients, reporting percentages of occurrence with corresponding 95% confidence intervals. With n = 15, the precision of an exact 95% confidence interval ranges from 11% to a maximum of 26% depending on the number of occurrences out of 15. In addition, we calculated that 8 patients would need to be administered the treatment without occurrence of an SAE to conclude the drug is safe with 95% confidence, considering ‘safe’ as the probability of an SAE is less than 40%. The limit of 40% was selected based on earlier clinical trials reporting on the frequency of treatment-related SAEs for atezolizumab and platinum-based chemotherapy combination treatments for other malignancies [35, 36].

Feasibility and safety measures will be reported as percentage with 95% Clopper-Pearson confidence interval (CI). In addition, the number and grade of AEs and SAEs will be summarized. The Immuno-MESODEC study will be considered successful from a feasibility and safety perspective when >66% of included patients complete the study treatment schedule and when ≤ 40% of the evaluable patients experienced atezolizumab- or WT1/DC-related SAEs within 6 months after initiating first-line treatment, respectively.

For the assessment of clinical activity, BOR and DOR will be determined for each evaluable patient and summarized. DCR and ORR proportions will be reported with corresponding 95% Clopper-Pearson CI. For the survival measures, PFS and OS, median times (or lower bound) with 95% CI and Kaplan-Meier curves will be determined. Immunomonitoring data for evaluable patients will be summarized, parameters at each time point will be compared and associations with clinical data and survival will be examined.

For QoL evaluation for the evaluable population, summary scores will be calculated according to the questionnaires’ manuals and a plot of QoL scores over time will be considered. Any relationships with clinical data will be studied graphically by visual inspection of plotted data to identify trends and association measures will be calculated. For biomarker evaluation, associations between the investigated immunological and tumor parameters as well as with clinical data, immunological responses and survival will be studied graphically and if homogeneity of population allows, association measures can be calculated.

Data will be analyzed at three time points: (i) when 8 patients have survived 24 months after diagnosis, (ii) when all patients have reached an event (i.e. death) or 24 months after diagnosis and (iii) at end of study. If the time between the second and final analysis is expected to be less than 12 months, only final analysis at end of study will be performed.

## Strengths, limitations & future perspectives

The Immuno-MESODEC study is unique in its kind, integrating two types of immunotherapy into the standard chemotherapy-based first-line treatment regimen for hard-to-treat epithelioid MPM. This trial is designed to yield essential information on the safety and feasibility of the proposed treatment regimen. While analysis of efficacy will also be performed, these results will need to be interpreted with caution, given the small sample size and the absence of robust comparative data in the form of a study-inherent control arm. In any case, results of this phase I/II trial will be useful to guide the further implementation trajectory for DC-based immunotherapy in clinical practice for MPM treatment. Comparing these results against those of historical patient cohorts treated with available standard-of-care treatments, as well as those of the MESODEC trial (NCT02649829), in which patients were treated with chemotherapy plus WT1/DC immunotherapy in first-line, and–in most cases—WT1/DC immunotherapy plus checkpoint inhibition in next-line, will allow us to estimate the effect size and design a larger scale, randomized controlled trial with the aim of delivering definite proof of the efficacy of WT1-targeted DC immunotherapy in combination with PD-L1-targeted checkpoint inhibition and chemotherapy in an optimal configuration for treating epithelioid MPM.

## Trial status

The Immuno-MESODEC study has been approved by the Belgian competent authorities (FAMHP) and the Ethics Committee in October 2022 and in December 2022, respectively. Recruitment has been opened on February 24^th^, 2023 and is expected to be completed by the end of 2024. The current study protocol is version 1.5, dated June 15, 2023. Results will be published in peer-reviewed journals and presented at international conferences. End of trial summaries will appear on regulatory authority databases and a summary of the results will be provided to recruiting sites so that participants are able to access the results via their treating clinician.

## Supporting information

S1 File Clinical trial protocol v1.5. (PDF)

S2 File SPIRIT checklist. (PDF)
