# Supplementary material for: Integration of the PD-L1 inhibitor atezolizumab and WT1/DC vaccination into standard-of-care first-line treatment for patients with epithelioid malignant pleural mesothelioma—Protocol of the Immuno-MESODEC study
Source: PLoS One. 2024 Jul 15;19(7):e0307204. doi: 10.1371/journal.pone.0307204 (PMC11249236; doi:10.1371/journal.pone.0307204)
Supplement: S1 File — (PDF) [file pone.0307204.s001.pdf]

Antwerp University Hospital (UZA)
Drie Eikenstraat 655
B-2650 Edegem
BELGIUM

Center for Cell Therapy and Regenerative Medicine (CCRG)

Integration of the PD-L1 inhibitor atezolizumab and WT1/DC vaccination into platinum/pemetrexed-based first-line treatment for epithelioid malignant pleural mesothelioma

Immuno-MESODEC clinical trial

Internal protocol code: CCRG21-001
EudraCT number: 2021-003229-31
ClinicalTrials.gov: To be determined

Coordinating investigator **Zwi N. Berneman, MD, PhD**
Division of Hematology
Medical Director Center for Cell Therapy and Regenerative Medicine (CCRG)
Antwerp University Hospital (UZA)
[redacted]

Protocol version	Date of ethics committee approval
**v1.0 June 30, 2021**	N.A.
**v1.1 August 19, 2021**	N.A.
**v1.2 June 20, 2022**	N.A.
**v1.3 August 2, 2022**	05/12/2022
**v1.4 April 11, 2023**	N.A.
**v1.5 June 15, 2023**	21/08/2023

Table of contents

1 Protocol summary 5
2 Background and introduction 7
2.1 Malignant pleural mesothelioma 7
2.2 Treatment for MPM 7
2.2.1 Conventional treatments for MPM 7
2.2.2 Immunotherapy for MPM 7
2.2.3 Combination treatments for MPM 9
3 Objectives 11
3.1 Primary objectives 11
3.2 Secondary objectives 11
3.3 Exploratory objectives 11
4 Study population 12
4.1 Inclusion criteria 12
4.2 Exclusion criteria 13
5 Trial design 15
5.1 Overview of study design 15
5.2 Study-related procedures and treatment regimen 16
5.2.1 Patient screening and inclusion 16
5.2.2 DC vaccine manufacturing 16
5.2.3 DC vaccination 17
5.2.4 Evaluation of endpoints 19
5.3 Study schedule 22
5.3.1 Study scheme 22
5.3.2 Study calendar 23
6 Treatment information, expected toxicity and modifications 26
6.1 Chemotherapy information 26
6.2 Atezolizumab information 26
6.2.1 Nature of the therapy 26
6.2.2 Pharmaceutical data 30
6.2.3 Dose modification, delay or discontinuation 32
6.3 Vaccine information 32
6.3.1 Nature of the therapy 32
6.3.2 Pharmaceutical data 33
6.3.3 Vaccine dose omission or discontinuation 34
7 Response evaluation 36
7.1 Definition of evaluable patients 36
7.2 Analyses of responses 36

7.2.1 Feasibility and safety (Primary objective) 36
7.2.2 Clinical efficacy (Secondary and exploratory objectives) 36
7.2.3 Immunological responses and biomarker analysis (Secondary and exploratory objectives) 37
8 Safety evaluation 38
8.1 Definitions 38
8.1.1 Adverse event 38
8.1.2 Serious Adverse event 38
8.1.3 Adverse events of special interest 38
8.2 Severity of adverse events 38
8.3 Relationship of adverse events to study agents 39
8.4 Reporting procedures 40
8.5 Events or outcomes not qualifying as SAEs 40
9 Statistical considerations 41
9.1 Sample size and power calculations 41
9.2 Statistical analyses 41
9.2.1 Patient characteristics and baseline comparisons 41
9.2.2 Analysis of primary objectives 42
9.2.3 Analysis of secondary objectives 42
9.2.4 Analysis of exploratory objectives 42
9.3 Analyses and reporting 42
10 Administrative matters 43
10.1 Study documentation 43
10.1.1 Investigator records 43
10.1.2 Electronic case report forms 43
10.2 Monitoring procedures 43
10.3 Trial insurance 44
10.4 Publication policy 44
11 Ethical considerations 45
11.1 Patient protection 45
11.2 Informed consent 45
12 Organization of the study 46
12.1 Trial sponsorship and project funding 46
12.2 Organization of the study - overview 46
12.3 Clinical coordination 46
12.3.1 Coordinating investigator 46
12.3.2 Principal investigators 47
12.3.3 Subinvestigator 47
12.3.4 Study coordinators 47
12.4 Patient recruitment 48

12.5 Data management ... 48
12.6 Leukapheresis unit ... 49
12.7 Vaccine manufacturing ... 49
12.8 Vaccine preparation site ... 49
12.9 Vaccination site ... 49
12.10 Platinum/pemetrexed-based chemotherapy and atezolizumab administration ... 50
12.11 Immunomonitoring sites ... 50
12.12 Data monitoring ... 50
12.13 Statistical analyses ... 50
13 References ... 52
14 Appendices ... 55
14.1 Appendix A: Laboratory tests ... 55

1 Protocol summary

**Title of the study**	Integration of the PD-L1 inhibitor atezolizumab and WT1/DC vaccination into platinum/pemetrexed-based first-line treatment for epithelioid malignant pleural mesothelioma
**Study design**	Single arm, open-label, phase I/II trial
**Study population**	15 treatment-naïve unresectable malignant pleural mesothelioma (MPM) patients, epithelioid subtype (stage I-IV)
**Investigational treatment**	Atezolizumab (1200-1680 mg), administered as an intravenous (IV) infusion.<br>*Wilms' tumor 1 (WT1)* mRNA-electroporated dendritic cell (WT1/DC) vaccine (8-10 x 10^6 cells), administered through intradermal injection
**Objectives of the study**	*Primary objective*<br>▪ To investigate the <u>feasibility and safety</u> of adding the programmed death-ligand 1 (PD-L1) inhibitor atezolizumab and WT1/DC vaccination to first-line platinum/pemetrexed-based chemotherapy in patients with epithelioid MPM<br>*Secondary objectives:*<br>▪ To assess <u>indicators of clinical activity</u> of first-line platinum/pemetrexed-based chemotherapy when combined with atezolizumab and WT1/DC vaccination in epithelioid MPM patients<br>▪ To determine the <u>immunogenicity</u> of atezolizumab and WT1/DC vaccination when added to first-line platinum/pemetrexed-based chemotherapy in epithelioid MPM patients<br>*Exploratory objectives:*<br>▪ To assess <u>clinical efficacy</u> of next-line treatments in epithelioid MPM patients, following or in combination with WT1/DC vaccination<br>▪ To screen for <u>prognostic, predictive and/or therapeutic biomarkers</u><br>▪ To evaluate general <u>quality of life and disease-related symptoms</u> using EQ-5D-5L and LCSS-Meso questionnaires over the treatment course, including association with clinical outcome
**Study treatment scheme**	*Intervention and study treatment scheme:*<br>▪ Leukocyte apheresis to harvest peripheral blood mononuclear cells (PBMCs) to produce WT1/DC vaccines<br>▪ Treatment with four 3-weekly (±3 days) platinum/pemetrexed-based chemotherapy cycles combined with atezolizumab treatments and WT1/DC vaccinations at day 0 and day 14 (±3 days) of each chemotherapy cycle, respectively.<br>*Continuation of atezolizumab treatment and/or WT1/DC vaccination:*<br>Additional atezolizumab doses and/or WT1/DC vaccines after the chemo-immunotherapy study scheme can be administered to the patient if consent for continuation of atezolizumab treatment and/or WT1/DC vaccination was obtained and residual WT1/DC vaccine aliquots are available. In that case, atezolizumab and/or WT1/DC vaccines will be administered on a 4-weekly basis (±1 week). The WT1/DC vaccines will be administered within 1 week after atezolizumab administration.

**Follow-up**	After the final WT1/DC vaccination and/or atezolizumab administration, patients will enter a follow-up phase that lasts for up to 90 days after final WT1/DC vaccination and/or atezolizumab administration or 24 months after diagnosis, whichever occurs later.
**Major inclusion criteria**	▪ Signed informed consent<br>▪ Diagnosis with histologically proven epithelioid unresectable MPM (stage I-IV)<br>▪ Aged ≥18 years at the time of signing the informed consent form<br>▪ World Health Organization (WHO) performance status: grade 0-1<br>▪ Adequate hematologic and end-organ function<br>▪ Negative viral serology for Human Immunodeficiency Virus (HIV), Hepatitis B Virus (HBV) or Hepatitis C Virus (HCV)<br>▪ Willing and able to comply with the study protocol, as judged by the treating physician<br>▪ Women of childbearing potential must have a negative serum or urine pregnancy test at the time of screening.
**Major exclusion criteria**	▪ History of another malignancy within the last three years (except for malignancies with a negligible risk of metastasis or death)<br>▪ Symptomatic, untreated, or actively progressing central nervous system metastases<br>▪ Active or history of autoimmune disease or immune deficiency<br>▪ Severe infection within 4 weeks prior to initiation of study treatment<br>▪ Prior treatment for MPM<br>▪ Prior allogeneic stem cell or solid organ transplantation<br>▪ Major surgical procedure, other than for diagnosis, within 4 weeks prior to initiation of study treatment, or anticipation of need for a major surgical procedure during the study<br>▪ Use of any investigational agent within 28 days before study enrollment<br>▪ Recent treatment with systemic immunostimulatory agents or systemic immunosuppressive medication<br>▪ Pregnant or breastfeeding<br>▪ Any other condition, either physical or psychological, or reasonable suspicion thereof on clinical or special investigation, which contraindicates the use of atezolizumab, pemetrexed, cisplatin/carboplatin and/or WT1/DC vaccines, or may negatively affect patient compliance, or may place the patient at higher risk of potential treatment complications.

2 Background and introduction

2.1 Malignant pleural mesothelioma

Malignant pleural mesothelioma (MPM) – an aggressive neoplasm arising from the mesothelial surfaces of the pleural cavities – is a highly aggressive and in virtually all cases fatal cancer that is tightly associated with prior asbestos exposure.[1] Although preventive measures to limit asbestos use and exposure have been around for several decades, the incidence of MPM is only expected to decrease very slowly for several years ahead due to the long latency between asbestos exposure and MPM development and the persistence of environmental exposure.[2,3]

2.2 Treatment for MPM

2.2.1 Conventional treatments for MPM

First-line combination chemotherapy, consisting of a platinum compound and the folate antimetabolite pemetrexed, has been the approved standard-of-care since 2004, resulting in a dismal median overall survival (OS) from diagnosis of only 9-16 months, depending on the histological subtype.[1,4-6] Perhaps the most important reason for this continued poor outcome is that conventional anti-cancer treatments (i.e. chemotherapy, surgery and radiotherapy) have very limited effectiveness in MPM.[2,7]

2.2.2 Immunotherapy for MPM

The immune system constitutes a natural defense mechanism against cancer. Tumors, however, employ a variety of immune evasive mechanisms to allow their growth and expansion. As a consequence, an intricate interplay between anti-tumor immunity and a pro-tumoral immunosuppressive microenvironment arises, the balance of which contributes to tumor control or progression, respectively. The goal of immunotherapy is to tilt this balance by reinforcing, stimulating and/or arming the body's own immune system to establish a more vigorous anti-tumor immune activation.

2.2.2.1 *Checkpoint inhibition*

Recently, the first significant and clinically meaningful improvement in unresectable MPM prognosis with immunotherapy versus standard-of-care platinum/pemetrexed-based chemotherapy has been reported in the 'Checkmate 743' study. After a median follow-up of 29.7 months, a durable survival benefit was observed for epithelioid MPM patients treated in first-line with immunotherapeutic monoclonal antibodies inhibiting the programmed cell death protein-1 (PD-1) (nivolumab, OPDIVO®) and cytotoxic T-lymphocyte-associated protein 4 (CTLA-4) (ipilimumab, YERVOY®) (median OS 18.7 months [95% confidence interval (CI) 16.9-22.0]), as compared to patients treated with standard-of-care chemotherapy (mOS 16.5 months [95% CI 14.9-20.5]). For the non-epithelioid subtype, the treatment effect was even more pronounced (median OS 18.1 months [95% CI 12.2–22.8] as compared to 8.8 months [95% CI 7.4–10.2] for standard-of-care chemotherapy).[8] Based on these results, this particular combinational checkpoint inhibition treatment regimen was approved by the United States of America (USA) Food and Drug Agency (FDA) and the European Medicines Agency (EMA) as an alternative standard-of-care first-line treatment for unresectable MPM. Hence, immunotherapy for MPM treatment is at a pivotal point and combinatorial treatment approaches aiming to correct the immunological imbalance associated with MPM could pave the way to substantially improved patient outcomes.

2.2.2.2 *DC vaccination*

Owing to their status as most effective professional antigen-presenting cells and key orchestrators of the immune system[9], dendritic cells (DC) have claimed central stage in the development of active cancer immunotherapy over recent decades[10,11]. DC act as potent activators of natural killer (NK) cells and CD8+ T cells, which, as key effector cells of the innate and adaptive immune system, respectively,

are capable of eradicating malignant cells. In this way, DC immunotherapy could contribute to the establishment of long-lasting antitumor immunity, thereby improving the patient's outcome.

Since the publication of the first clinical trial in 1996[12], DC vaccination was repeatedly shown to be safe and well-tolerated, with side-effects generally being limited to local injection site reactions. Moreover, these studies as well as our own clinical data have demonstrated that DC vaccination is capable of inducing immunological and clinical responses in patients with different hematological and solid malignancies[11,13-16].

2.2.2.2.1 Wilms' tumor 1-targeting DC vaccination

The concept of antigen-specific vaccination against MPM is based on the notion that mesothelioma cells, like many other cancer cells, carry tumor-associated antigens (TAAs) allowing their recognition and elimination by cytotoxic T lymphocytes (CTLs).[17] Several TAA have been identified in malignant mesothelioma cells,[18] including the Wilms' tumor antigen 1 (WT1) which is overexpressed in up to 99% of all MPM cases.[19] Our own clinical experience with autologous WT1/DC vaccination in patients with different hematological and solid malignancies, including MPM patients, further underscores the safety of WT1/DC vaccination.[15,16,20]

2.2.2.2.2 WT1/DC vaccination for MPM

In a first phase I/II clinical trial (ClinicalTrials.gov: NCT01291420), we evaluated the feasibility, safety, immunogenicity and clinical efficacy of WT1/DC vaccination as consolidation treatment following standard combination chemotherapy in patients with inoperable epithelioid MPM. Ten patients (median age 62 [range: 53-73]) with non-progressive disease after frontline chemotherapy underwent leukapheresis. Clinical-grade autologous DC vaccines, engineered to express WT1 through mRNA electroporation – a highly efficient and clinically applicable antigen delivery method that was pioneered more than one decade ago in our laboratory[20,21] – were prepared as previously described,[14] with a novel *WT1* mRNA construct optimized for enhanced antigenic expression and T cell presentation.[22] Biweekly intradermal vaccinations were administered for an intended period of 6 months, followed by monthly or bimonthly injections until disease progression. Results from this study show that leukapheresis was successful in all patients, with a mean number of 19 WT1/DC vaccines produced (range: 7-29); 3 patients underwent an additional leukapheresis. A mean number of 18 vaccines were administered (range: 5-44). WT1/DC vaccination was well-tolerated and produced only mild and self-limiting local reactions at the injection sites. No systemic toxicity was observed. *In vivo* evidence of vaccine-elicited immunity was obtained in 9/10 patients. Median OS was 35.7 months after a median follow-up of 55.9 months post-start chemotherapy (31.8 months after a median follow-up of 55.9 after exclusion of 4 patients who were long-term survivors before DC vaccination); this compares favorably to the OS of 22 months reported in the literature for a similar cohort of patients.[23] The median progression-free survival (PFS), calculated from the time of registration in the study to the date of progression, was 4.95 months. These results suggest that adjuvant DC-based immunotherapy provides a clinical benefit for these patients.[16]

Following these encouraging results, a subsequent phase I/II trial (ClinicalTrials.gov: NCT02649829) was initiated to assess first-line combinatorial treatment of platinum/pemetrexed-based chemotherapy with WT1/DC vaccination for MPM patients. The overall aim of this clinical trial is to demonstrate that upfront combinatorial treatment of platinum/pemetrexed-based chemotherapy with WT1/DC vaccines is feasible and safe, improves patient's outcome and enables the induction of cancer-specific immune responses in patients with MPM. So far, local mild adverse reactions (ARs) related to WT1/DC vaccination (i.e. swelling, redness and itch at the injection site) have been reported regularly, as well as isolated cases of systemic mild ARs following WT1/DC vaccination (see Section 6.3.3 for more details). No serious adverse events (SAEs) possibly, probably or definitely related to the vaccine developed during the trial, suggesting that WT1/DC vaccination in combination with platinum/pemetrexed-based chemotherapy is well tolerated and confirming its overall beneficial safety profile (unpublished data, refer to the Investigator's Brochure for more details).
Moreover, preliminary data at the first analysis time point (dd. 27/10/2020) indicate a clinical benefit, as compared to recent published data of the CheckMate 743 study.[8] The best overall response (BOR) immediately after the 12-week chemo-immunotherapy treatment schedule in our ongoing clinical study was partial response (PR) in 11 patients (58%) and stable disease (SD) in 3 patients (16%). These preliminary results compare favorably with the reported objective response rate (ORR) of 43% in the

platinum/pemetrexed-based chemotherapy arm and 40% in the nivolumab plus ipilimumab arm of the CheckMate 743 study, respectively. In October 2020, 10 out of 20 enrolled patients had reached the 18-month follow-up landmark, with a preliminary median OS from diagnosis of 35 months (95%CI [24 months, not reached]), exceeding the median OS of 14-18 months reported in the CheckMate 743 study. Five patients died after a median follow-up period of 18 months from the time of diagnosis (range 13 - 35 months).
Despite these encouraging results, progression after the 12-week chemo-immunotherapy schedule was observed in 15 out of 20 patients (dd.27/10/2020). Based on the FAMHP-approved medical need program (MNP) for nivolumab in MPM (closed since March 2020), 13 among them were treated with nivolumab (q2w, 3 mg/kg) as second-line treatment, while WT1/DC vaccination was continued. One patient received nivolumab (q2w, 3 mg/kg) as third-line treatment along with continued WT1/DC vaccination. During combinatorial treatment of WT1/DC vaccination with the PD-1 inhibitor nivolumab, several patients presented with expected AR to nivolumab, such as anemia, diarrhea, rash, fatigue and pneumonitis. One patient developed a severe case of nivolumab-induced Guillain Barré syndrome, which was stabilized after plasmapheresis treatment. Importantly, no unexpected ARs were reported during the combination treatment of nivolumab and WT1/DC vaccination, which supports the novel combination strategy of PD1/PD-L1 targeted therapy and WT1/DC vaccination from a safety perspective. Preliminary data show complete response (CR) in 2 patients (15%), PR in 4 patients (31%) and SD in 3 patients (23%). The patient who received nivolumab as third-line treatment during WT1/DC vaccination is in CR since January 4, 2020. These results compare favorably with clinical trials investigating nivolumab monotherapy (i.e. MAPS-2[24] and NIVOMES study[25-27] and CONFIRM trial[26,27]) or/and nivolumab plus ipilimumab treatment (i.e. MAPS-2 and INITIATE study[28]) as next-line treatment for MPM, in which no CR was observed.

2.2.3 Combination treatments for MPM

2.2.3.1 *Combining DC vaccination with other treatment modalities*

Multiple arguments and accumulating lines of evidence are in support of the existence of a therapeutic synergism between DC vaccination and other (conventional) anti-mesothelioma treatments[29,30].

2.2.3.1.1 DC therapy and checkpoint inhibition

It is reasonable to assume that DC-induced immune responses can be hampered by an immunosuppressive tumor microenvironment. Checkpoint-inhibiting immunotherapies, on the other hand, typically require an ongoing T cell response at the tumor location to be effective. Combining both types of treatment thus could result in strong therapeutic synergism. Depending on the specific immune checkpoint pathway targeted, the mechanisms underlying synergism might be slightly different, but equally aim to break down the immunosuppressive tumor microenvironment. While CTLA-4-targeted checkpoint inhibition leads to regulatory T cell depletion at the tumor site and enhanced priming of naïve T cells in the lymph nodes, targeting the PD-1/ PD-L1 axis effectively reduces T-cell exhaustion and enhances T-cell effector functions. Moreover, since DC, including monocyte-derived DC, express both PD-L1[31] and PD-1[32], increased stimulatory capacity and survival of these cells under inhibition of the PD-1/PD-L1-axis can be anticipated[32-34]. Improved anti-tumor responses suggestive of reciprocal stimulatory effects have indeed been observed when combining either CTLA-4-[35], PD-1-[36] or PD-L1-[37] targeted checkpoint inhibition with DC vaccination in murine models and in *in vitro* human models[34,38,39].

2.2.3.1.2 DC therapy and chemotherapy

Chemotherapy and immunotherapy have historically been considered non-compatible treatment options, primarily due to the non-specific cytotoxic actions of the former. This long-standing paradigm is now crumbling. Increased antigen exposure following chemotherapy-induced immunogenic tumor-cell damage, the preferential elimination of regulatory cells by certain chemotherapeutics and the enhanced immune responsiveness due to stimulatory cytokines produced in response to cytotoxic immune- and tumor-cell damage[40], support a stimulatory role for chemotherapy in DC-induced anti-cancer immunity. Conversely, increased chemosensitivity after DC vaccination has also been reported in different types of cancer[41,42], but the mechanisms behind this phenomenon remain elusive.

2.2.3.1.3 Combinatorial treatment regimen aimed at therapeutic synergism in epithelioid MPM

Overall, it is becoming increasingly clear that the direct and indirect immunological actions of conventional and next-generation therapeutics are crucial in determining anti-cancer treatment outcome. The design of optimized combinatorial treatment paradigms, which maximally exploit the synergistic interactions between different classes of therapeutics, could further improve ORR. In this perspective, combinatorial approaches including DC vaccination are of particular interest, given its beneficial safety profile and fundamental immunological mode of action. Moreover, due to the longevity of the immune response, treatment synergy could outlast the period of DC administration.

Given the above outlined rationale, the anticipation on the encouraging preliminary data obtained in our ongoing clinical trial (ClinicalTrials.gov: NCT02649829) and supported by the recent evolutions in the cancer immunotherapy landscape, here, a new phase I/II multicentric clinical trial is initiated, where WT1/DC vaccination and the PD-L1-targeting immune checkpoint inhibitor atezolizumab are integrated in first-line chemotherapy-based treatment for MPM to further ameliorate patient outcome.

Since the epithelioid subtype of MPM has been shown to have an increased baseline sensitivity to chemotherapy as compared to the non-epithelioid subtype, appears to benefit less explicitly from checkpoint inhibition in monotherapy, but at the same time appears to be well responsive to WT1/DC vaccination (and subsequent checkpoint inhibitory therapy) as evident from our previous and ongoing clinical trials, the current study was designed for epithelioid MPM specifically.

3 Objectives

The general objective of this phase I/II clinical study is to demonstrate that addition of the PD-L1 inhibitor atezolizumab and WT1/DC vaccination to frontline conventional platinum/pemetrexed-based chemotherapy for the treatment of epithelioid MPM is feasible and safe. In addition, chemo-immunotherapy-induced immunogenicity will be studied and patient's clinical outcome will be documented for comparison with current patient's outcome allowing indication of the added value.

3.1 Primary objectives

- To investigate the feasibility and safety of adding atezolizumab and WT1/DC vaccination to first-line platinum/pemetrexed-based chemotherapy in patients with epithelioid MPM.

Refer to Section 7.2.1 for definition of the primary endpoints of this study.

3.2 Secondary objectives

- To assess indicators of clinical activity of first-line platinum/pemetrexed-based chemotherapy when combined with atezolizumab and WT1/DC vaccination in epithelioid MPM patients.

- To determine the immunogenicity of atezolizumab and WT1/DC vaccination when added to first-line platinum/pemetrexed-based chemotherapy in epithelioid MPM patients.

Refer to Section 7.2.2 for definition of the secondary endpoints of this study.

3.3 Exploratory objectives

- To assess clinical efficacy of next-line treatments in epithelioid MPM patients, following or in combination with WT1/DC vaccination.
- To screen for prognostic, predictive and/or therapeutic biomarkers.
- To evaluate general quality of life and disease-related symptoms using EQ-5D-5L and LCSS-Meso questionnaires over the treatment course, including association with clinical outcome.

Refer to Section 5.2.4.6 and 7.2.3 for definition of the exploratory endpoints of this study.

4 Study population

This open-label single arm, phase I/II study will include 15 evaluable patients diagnosed with epithelioid MPM.

4.1 Inclusion criteria

Subjects must meet all the following criteria to be eligible to participate in the study:

- Signed informed consent
- Diagnosis with histologically proven epithelioid unresectable MPM (stage I-IV)
- Age ≥ 18 years at the time of signing informed consent
- World Health Organization (WHO) performance status 0-1
- Adequate hematologic and end-organ function, defined by the following laboratory test results, obtained at the time of screening:
    - Absolute neutrophil count (ANC) ≥ 1.5 x 10^9/L (1500/µL) without granulocyte colony-stimulating factor support
    - Lymphocyte count ≥ 0.5 x 10^9/L (500/µL)
    - Platelet count ≥ 100 x 10^9/L (100,000/µL) without transfusion
    - Hemoglobin ≥ 90 g/L (9 g/dL)
      Patients may be transfused to meet this criterion
    - Aspartate aminotransferase (AST), alanine aminotransferase (ALT), and alkaline phosphatase (ALP) ≤ 2.5 x upper limit of normal (ULN), with the following exceptions:
        - Patients with documented liver metastases: AST and ALT ≤ 5 x ULN
        - Patients with documented liver or bone metastases: ALP ≤ 5 x ULN
    - Total bilirubin ≤ 1.5 x ULN with the following exception:
        - Patients with known Gilbert disease: total bilirubin ≤ 3 x ULN
    - Creatinine ≤ 1.5 x ULN
    - Albumin ≥ 25 g/L (2.5 g/dL)
    - For patients not receiving therapeutic anticoagulation: prothrombin international normalized ration (PT-INR) and activated partial thromboplastin time (APTT) ≤ 1.5 x ULN
- Negative Human Immunodeficiency Virus (HIV) test at screening
- Negative hepatitis B surface antigen (HBsAg) test at screening
- Negative total hepatitis B core antibody (HBcAb) test at screening, or positive total HBcAb test followed by a negative hepatitis B virus (HBV) DNA test at screening
    - The HBV DNA test will be performed only for patients who have a negative HBsAg test and a positive total HBcAb test.
- Negative hepatitis C virus (HCV) antibody test at screening, or positive HCV antibody test followed by a negative HCV RNA test at screening. The HCV RNA test must be performed for patients who have a positive HCV antibody test.
- Willing and able to comply with the study protocol, as judged by the treating physician
- Women of childbearing potential must have a negative serum or urine pregnancy test at the time of screening and agree to use effective contraception (<1% failure rate per year) before, during and for at least five months after the last atezolizumab administration or at least hundred days after the last WT1/DC vaccine administration (whichever takes longer). Men must agree to use effective

contraception before, during and for at least hundred days after the last study treatment administration.

4.2 Exclusion criteria

Subjects who fulfill any of the following criteria will not be eligible for admission into the study:

- History of malignancy within 3 years prior to initiation of study treatment, with the exception of the cancer under investigation in this study and malignancies with a negligible risk of metastasis or death (e.g., 5-year OS rate > 90%), such as adequately treated carcinoma *in situ* of the cervix, non-melanoma skin carcinoma, localized prostate cancer, ductal carcinoma *in situ*, or Stage I uterine cancer
- Symptomatic, untreated, or actively progressing central nervous system (CNS) metastases. Asymptomatic patients with treated CNS lesions are eligible, provided that all of the following criteria are met:
  - Measurable disease, per RECIST v1.1, must be present outside the CNS.
  - The patient has no history of intracranial hemorrhage or spinal cord hemorrhage.
  - The patient has not undergone stereotactic radiotherapy within 7 days prior to initiation of study treatment, whole-brain radiotherapy within 14 days prior to initiation of study treatment, or neurosurgical resection within 28 days prior to initiation of study treatment.
  - The patient has no ongoing requirement for corticosteroids as therapy for CNS disease.
  - If the patient is receiving anti-convulsant therapy, the dose is considered stable.
  - Metastases are limited to the cerebellum or the supratentorial region (i.e., no metastases to the midbrain, pons, medulla, or spinal cord).
  - There is no evidence of interim progression between completion of CNS directed therapy and initiation of study treatment.
  - Asymptomatic patients with CNS metastases newly detected at screening are eligible for the study after receiving radiotherapy and/or surgery, with no need to repeat the screening brain scan.
- History of leptomeningeal disease
- Active or history of autoimmune disease or immune deficiency, including, but not limited to, myasthenia gravis, myositis, autoimmune hepatitis, systemic lupus erythematosus, rheumatoid arthritis, inflammatory bowel disease, anti-phospholipid antibody syndrome, Wegener granulomatosis, Sjögren syndrome, Guillain-Barré syndrome, or multiple sclerosis, with the following exceptions:
  - Patients with a history of autoimmune-related hypothyroidism who are on thyroid replacement hormone are eligible for the study.
  - Patients with controlled Type 1 diabetes mellitus who are on an insulin regimen are eligible for the study.
  - Patients with eczema, psoriasis, lichen simplex chronicus, or vitiligo with dermatologic manifestations only (e.g., patients with psoriatic arthritis are excluded) are eligible for the study provided all of following conditions are met:
    - Rash must cover < 10% of body surface area
    - Disease is well controlled at baseline and requires only low-potency topical corticosteroids
    - No occurrence of acute exacerbations of the underlying condition requiring psoralen plus ultraviolet A radiation, methotrexate, retinoids, biologic agents, oral calcineurin inhibitors, or high potency or oral corticosteroids within the previous 12 months
- History of idiopathic pulmonary fibrosis, organizing pneumonia (e.g., bronchiolitis obliterans), drug-induced pneumonitis, or idiopathic pneumonitis, or evidence of active pneumonitis on screening

chest computed tomography (CT) scan. History of radiation pneumonitis in the radiation field (fibrosis) is permitted.

- Significant cardiovascular disease (such as New York Heart Association Class II or greater cardiac disease, myocardial infarction, or cerebrovascular accident) within 3 months prior to initiation of study treatment, unstable arrhythmia, or unstable angina
- Major surgical procedure, other than for diagnosis, within 4 weeks prior to initiation of study treatment, or anticipation of need for a major surgical procedure during the study
- Severe infection within 4 weeks prior to initiation of study treatment, including, but not limited to, hospitalization for complications of infection, bacteremia, or severe pneumonia, or any active infection that could impact patient safety
- Prior treatment for MPM
- Treatment with therapeutic oral or IV antibiotics within 2 weeks prior to initiation of study treatment. Patients receiving prophylactic antibiotics (e.g., to prevent a urinary tract infection or chronic obstructive pulmonary disease (COPD) exacerbation) are eligible for the study.
- Prior allogeneic stem cell or solid organ transplantation
- Use of any investigational agent within 28 days before study enrollment
- Pregnant or breastfeeding. Female subjects who are breastfeeding should discontinue nursing prior to the first dose of study treatment and until at least hundred days after the last study treatment administration.
- Treatment with a live, attenuated vaccine within 4 weeks prior to initiation of study treatment, or anticipation of need for such a vaccine during atezolizumab treatment or within 5 months after the final dose of atezolizumab.
- Current treatment with anti-viral therapy for HBV
- Prior treatment with CD137 agonists or immune checkpoint blockade therapies, including anti-CTLA-4, anti-PD-1, and anti-PD-L1 therapeutic antibodies
- Treatment with systemic immunosuppressive medication (including, but not limited to, corticosteroids, cyclophosphamide, azathioprine, methotrexate, thalidomide, and anti-tumor necrosis factor-α [TNF-α] agents) within 2 weeks prior to initiation of study treatment, or anticipation of need for systemic immunosuppressive medication during study treatment, with the following exceptions:
  - Patients who received acute, low-dose systemic immunosuppressant medication or a one-time pulse dose of systemic immunosuppressant medication (e.g., 48 hours of corticosteroids for a contrast allergy) may be eligible for the study after Medical Monitor confirmation has been obtained.
  - Patients who received mineralocorticoids (e.g., fludrocortisone), inhaled or low dose corticosteroids for COPD or asthma, or low-dose corticosteroids for orthostatic hypotension or adrenal insufficiency are eligible for the study.
- Treatment with systemic immunostimulatory agents (including but not limited to interferons or interleukin-2) within 4 weeks or 5 drug-elimination half-lives of the drug, whichever is longer, prior to initiation of study treatment
- History of severe allergic anaphylactic reactions to chimeric or humanized antibodies or fusion proteins
- Known hypersensitivity to Chinese hamster ovary cell products or to any component of the atezolizumab formulation
- Any other condition, either physical or psychological, or reasonable suspicion thereof on clinical or special investigation, which contraindicates the use of atezolizumab, pemetrexed, cisplatin/carboplatin and/or WT1/DC vaccination, or may negatively affect patient compliance, or may place the patient at higher risk of potential treatment complications.

5 Trial design

5.1 Overview of study design

Type of study. This is a single arm, open-label, phase I/II clinical trial for treatment-naïve unresectable epithelioid MPM patients to demonstrate that the combination of conventional platinum/pemetrexed-based chemotherapy with the PD-L1 inhibitor atezolizumab and WT1/DC vaccination is feasible and safe, enables the induction of mesothelioma-specific immune responses and improves patient outcome.

Type of intervention. Dual immunotherapy using the PD-L1 inhibitor atezolizumab and WT1/DCs combined with standard-of-care platinum/pemetrexed-based chemotherapy.

Study group. This study is intended to include 15 patients with treatment-naïve unresectable epithelioid MPM aged ≥18 years who are able to undergo leukapheresis, chemotherapy, immunotherapy and are fulfilling all other eligibility criteria *(refer to Section 4)*.

Start of study. The study is anticipated to start at the earliest in September 2022.

End of investigational treatment. End of investigational treatment is defined at the final administered (additional) WT1/DC vaccine and/or atezolizumab cycle. Reasons for end of treatment include:

- No more WT1/DC vaccines available
- Patient refuses continuation of WT1/DC vaccination and/or atezolizumab administration
- Unacceptable toxicity
- Major protocol violation
- Pregnancy

In addition, the Investigator may discontinue study treatment for a patient in the event of another illness, or for administrative or other reasons.

Follow-up. After the final WT1/DC vaccination and/or atezolizumab administration (i.e. end of investigational treatment), patients will enter a follow-up phase that lasts for up to 90 days after final WT1/DC vaccination and/or atezolizumab administration or 24 months after diagnosis, whichever occurs later. During this follow-up period, disease evaluation and survival follow-up must be continued according to the protocol. Following end of treatment due to the patient entering a terminal disease stage, patient follow-up can be performed as feasible, at the Investigator's discretion.

End of study (patient level). For the patient, the end of the study and the reasons thereof will be documented in the participant's case report form (CRF). Reasons for study end include:

- Ninety days after final WT1/DC vaccination and/or atezolizumab administration (i.e. end of investigational treatment) or 24 months after diagnosis, whichever occurs later (i.e. completion of study-related follow-up)
- Withdrawal of consent
- Lost to follow-up
- Death

The Investigator should only consider a patient lost to follow-up after documented attempts to reach the patient by phone (at least twice), e-mail and certified mail with return receipt.

All decisions and respective explanations must be documented in both the medical records and the CRF.

End of study (trial level). At the study level, end of study will occur when all evaluable patients have reached 90 days after final WT1/DC vaccination and/or atezolizumab administration (i.e. end of investigational treatment) or 24 months after diagnosis, whichever occurs later. The Sponsor has the right to monitor the patient's mesothelioma-related disease status (including imaging data) and anti-mesothelioma treatments, as well as to collect survival data beyond end of study as stated in the informed consent form.

Overview. A schematic overview of the study design and study calendar is depicted in section '5.3 - Study schedule'.

5.2 Study-related procedures and treatment regimen

5.2.1 Patient screening and inclusion

After signing of the informed consent form, patients will be evaluated for entry criteria during the screening period. All screening evaluations should be performed within 4 weeks before leukapheresis.

The following evaluations must be completed during the screening period:

- Eligibility screening according to the inclusion and exclusion criteria (*refer to Section 4)*.
  - Confirmation of diagnosis with histologically proven epithelioid unresectable MPM (stage I-IV) and eligibility to undergo chemotherapy and atezolizumab administration will be performed by the treating physician.
  - Eligibility to undergo leukapheresis and WT1/DC vaccination based on the patient's general medical condition and hematological blood values, and control of vascular accessibility will be confirmed by the responsible physicians of the Division of Hematology (UZA) and the Division of Nephrology (UZA), under the responsibility of the Cell and Tissue Bank (UZA).

  Eligibility screening is predominantly performed by the treating physician. In case of an incomplete screening visit, eligibility assessment can be completed at the Division of Hematology (UZA).
- Demographics and medical history
- Review of concurrent drug use
- Disease assessment:
  - Evaluation of the WHO performance status
  - CT scan with contrast of the chest and upper abdomen, if not already performed ≤ 4 weeks prior to start of the chemo-immunotherapy
  - CT/MRI brain
- General physical examination, including determination of vital signs (i.e. blood pressure, heart rate and temperature)
- Peripheral blood analysis (refer to Appendix A)

5.2.2 DC vaccine manufacturing

5.2.2.1 *Leukapheresis*

During the screening period, the data managers of the treating institution must contact the Center for Cell Therapy and Regenerative Medicine (CCRG) at UZA to plan a tentative date for leukapheresis to collect peripheral blood mononuclear cells (PBMCs) at the apheresis section of the Division of Nephrology, under the responsibility of the Cell and Tissue bank (UZA). CCRG must confirm the leukapheresis date to the GMP (Good Manufacturing Practices) manufacturing facility 1-3 days before it is performed.

Leukapheresis must be performed according to standard operating procedures (SOPs) at UZA using settings appropriate for monocyte collection. Patients must undergo leukapheresis prior to the first cycle of chemotherapy. Leukapheresis can be performed during the vitamin regimen which is required prior to the start of chemotherapy.

On the planned day of leukapheresis, the patient's differential blood count and hemostasis are checked and should be adequate for undergoing a leukapheresis procedure according to institutional guidelines. The value for monocyte count should conform to the criterion specified in the latest version of the IMPD. The patient's blood group and rhesus factor are tested. In addition, blood sampling for serology testing for HIV, HBV, HCV and syphilis is performed in line with the Royal Decree of 28 September 2009 concerning banks of human body material. A differential blood count is again performed after the leukapheresis procedure.

5.2.2.2 *DC generation*

$CD14^+$ monocytes will be isolated from the PBMC fraction using magnetic bead-labeled anti-CD14 monoclonal antibodies with the CliniMACS® Cell Separation System (Miltenyi Biotech, Germany) and subsequently used for *ex vivo* differentiation into immature DCs in the presence of 80 ng/mL [800-1060 IU/mL] granulocyte macrophage-colony stimulating factor and 250 IU/mL interleukin-4. DC cultures will be maintained in CellGenix® GMP DC Medium supplemented with 1% pre-tested human AB serum. At day 6, immature DCs will be matured through addition of 20 ng/mL tumor necrosis factor 〈, 2.5 µg/mL prostaglandin E2 and 10 µg/mL pyrogen-free keyhole limpet hemocyanin as a CD4+ T cell helper antigen. At day 8, mature DCs will be harvested and washed for downstream antigen loading through electroporation.

For other details, see also the latest version of the Investigational Medicinal Product Dossier (IMPD).

5.2.2.3 *Antigen loading of DCs*

Mature DCs are harvested, washed and resuspended in sterile electroporation medium (phenol red-free Opti-MEM, Gibco-Life Technologies, Merelbeke, Belgium). Full-length *WT1-DC-lysosomal associated membrane protein (LAMP)* mRNA has been engineered starting from the pGEM/WT1/A64 plasmid followed by optimization and subcloning in a DC-LAMP-containing vector for the production of clinical-grade *WT1-DC-LAMP* mRNA. Per electroporation, up to 50.10^6 DCs are electroporated with 20 µg *WT1-DC-LAMP* mRNA using a GenePulser Xcell electroporation device (Bio-Rad, Ghent, Belgium) according to the specifications in the latest version of the IMPD. Immediately after electroporation, cells are allowed to recover for 2 hours. *For other details, see also the latest version of the IMPD.*

5.2.2.4 *Cryopreservation, packaging and labeling of WT1/DCs*

Electroporated DCs are harvested and cryopreserved in vaccine aliquots in pre-tested human AB serum containing 10% dimethyl sulfoxide (DMSO) and 2% (w/v) glucose, at temperatures below -130°C.

Packaging and labeling of the cryopreserved DC vaccines will be in accordance with GMP for clinical trials and the Quality Manual of the GMP manufacturing facility, where records will be maintained. An inventory of all vaccines produced per included subject will be established, including all relevant information related to the production. The inventory must be available for monitoring, auditing or inspection.

For other details, see also the latest version of the IMPD.

5.2.3 DC vaccination

5.2.3.1 *DC vaccine release criteria*

The cryopreserved DC vaccine will be under embargo from release until the quality control test results have become available and all release criteria have been met. A detailed overview of all applicable release criteria is provided in the latest version of the IMPD. The embargo period generally lasts 3 weeks counting from the day of cryopreservation (i.e. 8-9 days after leukapheresis).

5.2.3.2 *DC vaccine preparation*

On the day of vaccination, one vial of pre-aliquoted WT1/DCs is thawed, according to the protocol specified in the latest version of the reconstitution handling manual. After thawing, the WT1/DC vaccine preparation is administered to the patient no later than 3 hours following the end of the preparation procedure after thawing of the cryopreserved DCs.

WT1/DC vaccines will be administered at UZA (8-10 x 10^6 cells in 500 µL saline solution with 5% human albumin) through intradermal injection at 5 sites (100 µL/site) in the ventromedial region of the upper arm (5-10 cm from the axillary lymph nodes). Injection sites will alternate between left and right arms.

For more details, see also the latest version of the reconstitution handling manual.

5.2.3.3 *Study treatment schedule*

The intention of the study is to administer four 3-weekly (±3 days) platinum/pemetrexed-based chemotherapy cycles (CT_{1-4}) in combination with four atezolizumab treatments (A_{1-4}) and four WT1/DC vaccinations (V_{1-4}) at day 0 and day 14 (±3 days) of each chemotherapy cycle, respectively.

The treating physician, in agreement with the Investigator, can at any time decide to discontinue treatment temporarily or indefinitely if deemed necessary in the benefit of the participant.

5.2.3.4 *Continuation of atezolizumab treatment and/or DC vaccination*

While the study treatment schedule consists of four chemo-immunotherapy cycles *(see Section 5.2.3.3)*, additional atezolizumab doses and/or WT1/DC vaccinations can be administered (optionally), on the conditions that:

- consent of the participant concerned was obtained, and
- residual WT1/DC vaccine aliquots are available.

Additional atezolizumab doses will be administered on a 4-weekly basis (±1 week), followed by a WT1/DC vaccination within one week, if available. Atezolizumab will be administered until disease progression or until unacceptable toxicity occurs or if the Investigator or participant decide to withdraw from therapy. WT1/DC vaccinations will be administered as long as residual vaccine aliquots are available or until unacceptable toxicity develops or if the Investigator or participant decide to withdraw from therapy.
In case atezolizumab administrations are discontinued, WT1/DC vaccination alone will be administered at 4-weekly intervals (±1 week).
In case of exhaustion of the WT1/DC vaccines, atezolizumab treatment alone can be continued at 4-weekly intervals (±1 week).

The treating physician, in agreement with the Investigator, can at any time decide to discontinue treatment temporarily or indefinitely if deemed necessary in the benefit of the participant.

5.2.3.5 *Treatment beyond (radiographical) disease progression*

In case of disease progression, the study treatment schedule will be discontinued. Patients will be treated at the Investigator's discretion based on availability of treatment options, including continuation of WT1/DC vaccination, and in agreement with the patient.

Treatment with atezolizumab should be discontinued in all patients who exhibit evidence of disease progression per RECIST v1.1. However, because of the possibility of an initial increase in tumor burden caused by immune-cell infiltration ("pseudoprogression") and because MPM patients whose disease progresses after first-line treatment have limited treatment options, and such options have limited efficacy and significant toxicity, patients may be considered for treatment with atezolizumab beyond radiographic disease progression per RECIST v1.1, in the absence of unacceptable toxicity, at the discretion of the Investigator and after appropriate discussion with the patient and obtaining informed consent. Patients will be permitted to continue atezolizumab if they meet all of the following criteria:

- Evidence of clinical benefit, as determined by the Investigator following a review of all available data
- Absence of symptoms and signs (including laboratory values) indicating unequivocal progression of disease
- Absence of a decline in WHO performance score that can be attributed to disease progression
- Absence of tumor progression at critical anatomical sites (e.g., leptomeningeal disease) that cannot be managed by protocol-allowed medical interventions

Patients who continue atezolizumab treatment beyond radiographic disease progression per RECIST v1.1 should be closely monitored clinically and with a follow-up scan in 6 weeks or sooner if symptomatic

deterioration occurs. Treatment should be discontinued if clinical deterioration due to disease progression occurs at any time, or if persistent disease growth is confirmed in a follow-up scan. In addition, patients should be discontinued for unacceptable toxicity or for any other signs or symptoms of deterioration attributed to disease progression as determined by the Investigator after an integrated assessment of radiographic data and clinical status.

During study participation, detailed information about all subsequent lines of therapy as well as survival data must be collected beyond disease progression for all patients.

The treating physician, in agreement with the Investigator, can at any time decide to discontinue treatment temporarily or indefinitely if deemed necessary in the benefit of the participant.

5.2.4 Evaluation of endpoints

5.2.4.1 *Patient evaluation (primary endpoint)*

Patient evaluation will be performed during regular clinic visits.

Time points:

- during the screening period
- at each chemotherapy (CT_1 to CT_4)/atezolizumab (A_1 to A_4) treatment visit
- at each vaccination visit (V_1 to V_4)
- at each additional atezolizumab treatment visit (A_x) or each third vaccination visit (V_x) (when additional WT1/DC vaccination alone)
- at final tumor response evaluation visit (FTEV)
- at follow-up visits (i.e. until 90 days after final WT1/DC vaccination and/or atezolizumab administration or 24 months after diagnosis, whichever occurs later).

Patient evaluation will include:

- General physical examination (Complete physical examination during screening. Limited, symptom-directed physical examination during subsequent visits and as clinically indicated), evaluation of vital signs (i.e. blood pressure, heart rate and temperature), WHO performance status, review of concurrent drug use and adverse event (AE) reporting. AEs are defined and graded according to the latest version of the National Cancer Institute's Common Terminology Criteria for Adverse Events (CTCAE) and Common Toxicity Criteria (CTC) *(at the time points described above)*.
  For more details, see: http://ctep.cancer.gov/protocolDevelopment/electronic_applications/ctc.htm
- Peripheral blood analysis *(at following time points: during the screening period, at each chemotherapy visit (CT_1 to CT_4) and/or atezolizumab treatment visit (A_1 to A_x) and FTEV and at the Investigator's discretion during follow-up visits) (refer to Appendix A)*

In case WT1/DC vaccination is continued without atezolizumab administration, patient evaluation and peripheral blood analysis will be performed at each third additional vaccination visit (V_x). If a next-line treatment with continued WT1/DC vaccination is started, patient evaluation and peripheral blood analysis will be performed at the day of administration of the next-line treatment.

All concurrent drug use should be reported in the patient's CRF during first-line treatment (including continuation of atezolizumab administration and/or WT1/DC vaccination) until 90 days after last investigational treatment. During next-line treatments with continuation of WT1/DC vaccination, only concomitant medication taken in the context of an AE that is probably, possibly or definitely related to WT1/DC vaccination or the combination of WT1/DC vaccination and a next-line anti-mesothelioma treatment should be reported in the patient's CRF until 90 days after final WT1/DC vaccination. For this, concurrent drug use at the time of occurrence of this AE as well as newly prescribed medication following this AE should be registered.

5.2.4.2 *Tumor evaluation (secondary endpoint)*

Tumor assessment will be performed.

Time points:

- ≤ 4 weeks before the start of the chemo-immunotherapy treatment (CT_1)
- Between CT_2 and CT_3
- After the fourth WT1/DC vaccination (V_4) (i.e. post CT_4)
- Within 12 weeks ± 2 weeks after V_4 (i.e. final tumor response evaluation visit, FTEV)
- At least every 12 weeks ± 1 week during the additional atezolizumab administration (A_x) and/or additional vaccination (V_x) period and during follow-up (i.e. until 90 days after final WT1/DC vaccination and/or atezolizumab administration or 24 months after diagnosis, whichever occurs later).
- ≤ 6 weeks following radiographical disease progression with continued atezolizumab administration, or sooner if symptomatic deterioration occurs.

Assessments should include a CT-scan with contrast or without contrast in case a CT-scan with contrast is contra-indicated of the chest and upper abdomen at the time points described above. An MRI scan at all tumor evaluation points may be used as an alternative.

5.2.4.3 *Indicators of clinical efficacy (secondary and exploratory endpoint)*

Patients will be followed for clinical response, disease progression and survival. Best overall response (BOR), duration of response (DOR) (for patients with an objective response only), objective response rate (ORR), disease control rate (DCR), PFS and OS for first-line platinum/pemetrexed-based chemotherapy in combination with atezolizumab and WT1/DC vaccination will be determined for all patients *(secondary endpoint)*. During study participation, next-line anti-mesothelioma treatments and accurate death date and the reason of death (cancer-related or non-related) will be recorded for every patient. Clinical efficacy of next-line treatments following or in combination with WT1/DC vaccination may be determined *(exploratory endpoint)*. Possible outcomes that may be considered are BOR, DOR, ORR, DCR, PFS and OS of next-line treatments. Follow-up after end of study (patient visit to the hospital or phone follow-up and frequency of tumor evaluation) will be done according to local practice. The Sponsor has the right to monitor the patient’s mesothelioma-related disease status (including imaging data) and anti-mesothelioma treatments as well as to collect survival data beyond end of study as stated in the informed consent.

5.2.4.4 *Systemic immunomonitoring studies (secondary endpoint)*

A blood draw for immunomonitoring studies will be performed prior to vaccination (baseline), after the chemo-immunotherapy treatment schedule and at the time of disease progression (if applicable). Therefore, patients will undergo blood sample collection (10x10 mL heparin tubes) at following time points:

- Before leukapheresis
- At the time of tumor evaluation after V_4 (i.e. post CT_4)
- At the time of disease progression, prior to the start of the second-line treatment (if applicable)

Patients with sustained disease control (stable disease (SD), partial response (PR) or complete response (CR)) for at least 12 months after the start of first-line chemo-immunotherapy will undergo an additional blood sample collection (10x10 mL heparin tubes) for immunomonitoring at 12 months (± 1 month) after the start of first-line chemo-immunotherapy.

All blood samples for immunomonitoring studies will be sent the same day to CCRG (UZA) and will be examined for, but not restricted to:

- Functional WT1-specific T-cell responses (e.g., WT1-reactive IFN-γ+ T cells)

5.2.4.5 *Tumor biopsy immunomonitoring studies (exploratory endpoint)*

Tumor biopsies obtained at diagnosis (if available) can be subjected to immunological assessments for biomarker screening.

5.2.4.6 *Patient-reported outcomes (exploratory endpoint)*

Patients will be asked to fill out general and disease-specific quality of life questionnaires (EQ-5D-5L and Lung Cancer Symptom Scale-Mesothelioma (LCSS-Meso)) to assess changes in general and disease-specific quality of life at the following time points:

- During the screening period
- Around the time of tumor evaluation between CT_2 and CT_3
- After the study treatment schedule, before additional atezolizumab administrations and/or WT1/DC vaccinations
- Around the time of tumor evaluation during additional atezolizumab administrations and/or WT1/DC vaccinations
- After the final atezolizumab administration or WT1/DC vaccination (whichever occurs latest), but before end of study (if possible)

The **EQ-5D-5L** is a generic measure of health status. The EQ-5D-5L consists of the EQ-5D descriptive system and the EQ visual analogue scale (EQ VAS).

- The EQ-5D descriptive system is a 5-item questionnaire that assesses 5 domains including mobility, self-care, usual activities, pain/discomfort and anxiety/depression. Each dimension has 5 levels: no problems, slight problems, moderate problems, severe problems and extreme problems. The patient is asked to indicate his/her health state by ticking the box next to the most appropriate statement in each of the five dimensions. This decision results in a 1-digit number that expresses the level selected for that dimension. The digits for the five dimensions can be combined into a 5-digit number that describes the patient's health state. Alternatively, the scores for the 5 dimensions are used to compute a single summary number (index value) which reflects how good or bad a health state is according to the preferences of the general population in a country/region.
- The EQ VAS is a visual analog scale rating "health today" with anchors ranging from 0 (worst imaginable health state) to 100 (best imaginable health state).The VAS can be used as a quantitative measure of health outcome that reflects the patient's own judgement.

The **LCSS-Meso** is a slightly modified version of the LCSS questionnaire, developed for use in mesothelioma patients. It includes five items measuring disease-related symptoms, including appetite loss, fatigue, cough, dyspnea and pain and three summary items related to total symptom burden, patient activity status and quality of life. The questionnaire uses a 24-hour recall period. Patient responses are captured using a 100-mm VAS per item. Each item is assigned and individual score equal to the length of the line marked by the patient, with 0 corresponding to the best rating and 100 representing the worst rating. The average of the aggregate score on all items is reported as the total score. In addition, a sub score representing the mean of all six major symptom scores (the average symptom burden index), the single global quality-of-life item, and/or individual items can be used to assess specific areas of change.

Endpoints for both quality of life questionnaires include, but are not restricted to:

- How patient disease-related symptoms evolve over time during the study
- How patient-reported general quality of life evolves over time during the study
- Associations between patient-reported disease-related symptoms and clinical outcome (i.e. clinical response, OS and PFS)
- Associations between patient-reported general quality of life and clinical outcome (i.e. clinical response, OS and PFS)

5.3 Study schedule

5.3.1 Study scheme

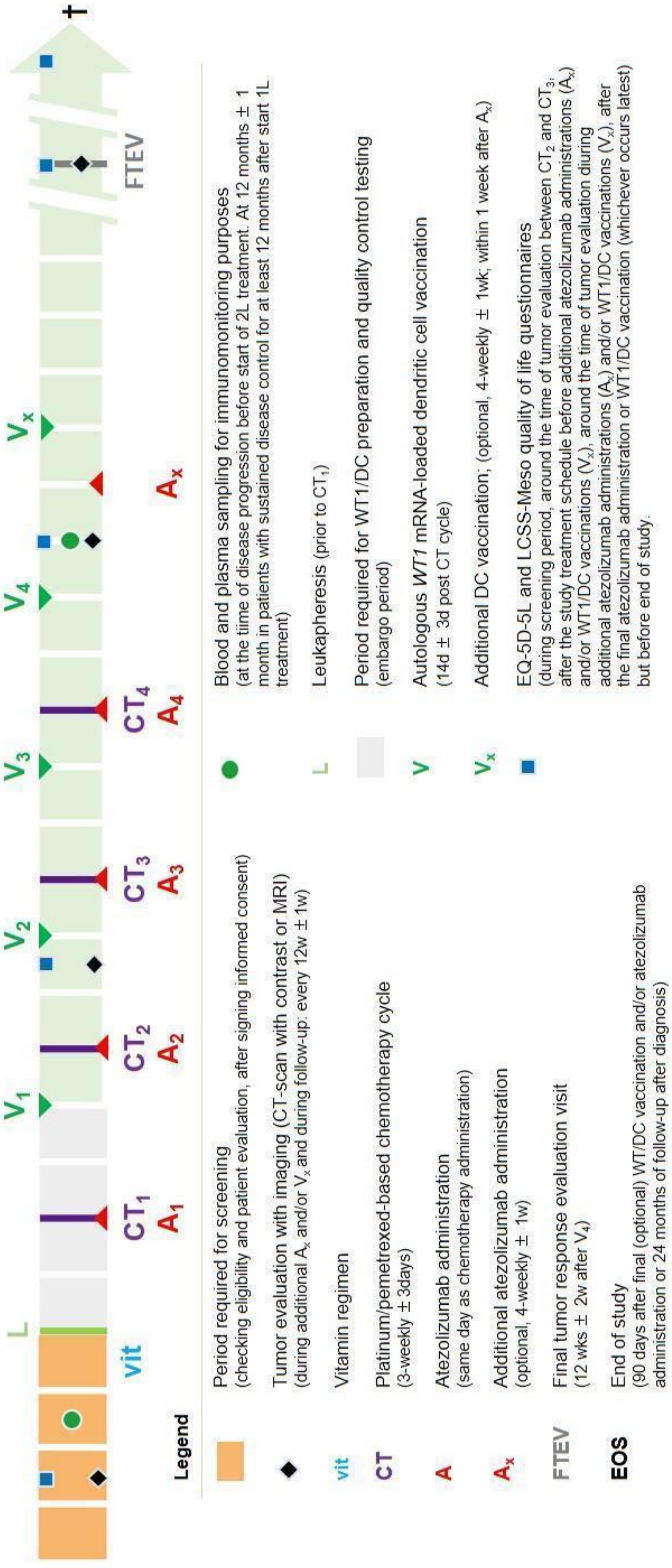

5.3.2 Study calendar

Time points used in the present study:

- Screening period (≤ 4 weeks before leukapheresis)
- L: time of leukapheresis
- CT_1: first chemotherapy cycle (after leukapheresis)
- A_1: first atezolizumab administration (at day 0 of CT_1)
- V_1: first WT1/DC vaccination (at day 14 ± 3 days of CT_1)
- CT_2: second chemotherapy cycle (3 weeks ± 3 days after CT_1)
- A_2: second atezolizumab administration (at day 0 of CT_2)
- V_2: second WT1/DC vaccination (at day 14 ± 3 days of CT_2)
- CT_3: third chemotherapy cycle (3 weeks ± 3 days after CT_2)
- A_3: third atezolizumab administration (at day 0 of CT_3)
- V_3: third WT1/DC vaccination (at day 14 ± 3 days of CT_3)
- CT_4: fourth chemotherapy cycle (3 weeks ± 3 days after CT_3)
- A_4: fourth atezolizumab administration (at day 0 of CT_4)
- V_4: fourth WT1/DC vaccination (at day 14 ± 3 days of CT_4)
- A_x/V_x: additional atezolizumab administrations and/or WT1/DC vaccinations, if available (optional, 4-weekly ± 1 week; vaccination within 1 week of atezolizumab administration)
- QoL: quality of life questionnaires (EQ-5D-5L and LCSS-Meso) during the screening period, around the time of tumor evaluation between CT_2 and CT_3, after the study treatment schedule before additional atezolizumab administrations and/or WT1/DC vaccinations, around the time of tumor evaluation during additional atezolizumab administrations and/or WT1/DC vaccinations and after the final atezolizumab administration or WT1/DC vaccination (whichever occurs later), but before end of study (if possible).
- FTEV: final tumor response evaluation visit (12 weeks ± 2 weeks after V_4). Subsequent tumor evaluations at least every 12 weeks ± 1 week during the additional atezolizumab (A_x) and/or additional vaccination (V_x) period and during follow-up until 90 days after the final WT1/DC vaccination and/or atezolizumab administration or 24 months after diagnosis, whichever occurs later)
- EOT: end of investigational treatment (variable, at final WT1/DC vaccination and/or atezolizumab administration V_x and/or A_x)
- EOS: Follow-up until 90 days after final WT1/DC vaccination and/or atezolizumab administration or 24 months after diagnosis, whichever occurs later.

Deviations from the outlined treatment schedule must be discussed with CCRG, the Data managers and the Investigator to ensure appropriate visit scheduling.

	Screening period	L	CT_1/A_1	V_1	CT_2/A_2	V_2	CT_3/A_3	V_3	CT_4/A_4	V_4	Post CT_4	A_x	V_x	FTEV	Follow-up[a]
Week (indicative only)	-4	0	2	4	5	7	8	10	11	13		15	16	25	
Eligibility	x														
WHO performance status[e]	x		x	x	x	x	x	x	x	x		x	(x)	x	x
Vital signs [e](i.e. blood pressure, heart rate and temperature)	x		x	x	x	x	x	x	x	x		x	(x)	x	x
Physical examination [e, g]	x		x	x	x	x	x	x	x	x		x	(x)	x	x
Medical history & demographics (incl. asbestos exposure and smoking history)	x														
Adverse event reporting		x	x	x	x	x	x	x	x	x	x	x	x	x	x
Concomitant medication [e]	x		x	x	x	x	x	x	x	x		x	(x)	x	x
CT with contrast or MRI of the chest and upper abdomen	x[b]					x					x	x[c]		x	x[c]
Quality of life questionnaires (EQ-5D-5L and LCSS-Meso)	x					x[d]					x[d]	x[d]		x[d]	x[d]
Peripheral blood analysis [e] 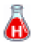	x	x	x		x		x		x			x	(x)	x	(x)
Peripheral blood analysis 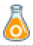	x	x													
Leukapheresis, including required donor screening		x													
Blood sampling for immunomonitoring studies (100 mL blood in heparin tubes)[f]	x										x				
Survival			x	x	x	x	x	x	x	x		x	x	x	x

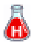 , hematology and biochemistry testing (refer to Appendix A)
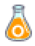 , serology, hemostasis, blood group, rhesus factor, pregnancy testing (refer to Appendix A)

[a], Until 90 days after final WT1/DC vaccination and/or atezolizumab administration or 24 months after diagnosis, whichever occurs later.

[b], Prior to the start of the chemotherapy treatment (CT_1). A CT-scan with contrast of the chest and upper abdomen must take place ≤ 4 weeks before the start of the chemotherapy treatment (CT_1). An MRI scan may be used as an alternative.

[c], A CT-scan with contrast or without contrast in case a CT-scan with contrast is contra-indicated of the chest and upper abdomen must take place at least every 12 weeks ± 1 week during the additional atezolizumab administration (A_x) and/or WT1/DC vaccination (V_x) period and during follow-up until 90 days after final WT1/DC vaccination and/or atezolizumab administration or 24 months after diagnosis, whichever occurs later. In case of radiographical disease progression with continued atezolizumab administration, a follow-up scan must be taken within 6 weeks, or sooner in case symptomatic deterioration occurs.

[d], Quality of life questionnaires (EQ-5D-5L and LCSS-Meso) during the screening period, around the time of disease assessment between CT_2 and CT_3, after the study treatment schedule but before additional atezolizumab administrations and/or WT1/DC vaccinations, around the time of tumor evaluation during additional atezolizumab administrations (A_x) and/or WT1/DC vaccinations (V_x) and after the final atezolizumab administration or WT1/DC vaccination (whichever occurs latest) but before end of study.

[e], In case WT1/DC vaccination is continued without atezolizumab administration, patient evaluation and peripheral blood analysis will be performed at each third vaccination visit (V_x) (i.e. (x) in table). If a next-line treatment with continued WT1/DC vaccination is started, patient evaluation and peripheral blood analysis will be performed at the day of administration of the next-line treatment. During follow-up, patient evaluation and peripheral blood analysis will be performed at Investigator's discretion (i.e. (x) in table). All concurrent drug use should be reported in the patient's CRF during first-line treatment (including continuation of atezolizumab administration and/or WT1/DC vaccination) until 90 days after last investigational treatment. During next-line treatments with continuation of WT1/DC vaccination, only concomitant medication taken in the context of an AE that is probably, possibly or definitely to WT1/DC vaccination or the combination of WT1/DC vaccination and a next-line anti-mesothelioma treatment should be reported in the patient's CRF until 90 days after final WT1/DC vaccination. For this, concurrent drug use at the time of occurrence of this AE as well as newly prescribed medication following this AE should be registered.

[f], Patients will undergo blood sample collection for immunomonitoring purposes before leukapheresis, at the time of tumor evaluation after V_4 (i.e. post CT4) and at the time of disease progression, prior to the start of the second-line treatment (if applicable). Patients with sustained disease control (stable disease (SD), partial response (PR) or complete response (CR)) for at least 12 months after the start of first-line chemo-immunotherapy will undergo an additional blood sample collection (10x10 mL heparin tubes) for immunomonitoring purposes at 12 months (± 1 month) after the start of first-line chemo-immunotherapy.

[g], General physical examination:

- Complete physical examination during screening
- Limited, symptom-directed physical examination during subsequent visits and as clinically indicated

6 Treatment information, expected toxicity and modifications

6.1 Chemotherapy information

In the context of this trial, the prescription of cisplatin/carboplatin and pemetrexed is considered as the standard-of-care first-line treatment. Chemotherapy, vitamin regimen and corticosteroids will be supplied and administered by the treating pulmonologist. Dose adjustments might be done as per standard procedures of the treating pulmonologist.

Pemetrexed (antifolate that inhibits four enzymes crucial for DNA synthesis) is licensed in combination with cisplatin (platinum compound that inhibits DNA synthesis) for the treatment of advanced/metastatic MPM.

On the first day of each cycle (day 0), pemetrexed 500 mg/m^2 should be administered as intravenous (IV) infusion over 10 minutes, followed by cisplatin 75 mg/m^2 as IV over approximately 2 hours. The actual doses of the drugs to be administered to patients will be determined by calculating the patient's body surface area at the beginning of each cycle. For ease of dose administration, the protocol allows ± 5% variance in the calculated total dose per infusion. If deemed necessary, the treating physician can decide to replace cisplatin by carboplatin. In that case, carboplatin will be delivered to an area under the concentration-time curve (AUC) of 5 as an IV infusion over 1 hour.

Chemotherapy will be administered for 4 doses or until unacceptable toxicity develops or if the Investigator or participant decides to withdraw from therapy.

Folic acid (350-600 µg) should be taken orally daily beginning at least one week before the first chemotherapy dose and should be continued until four weeks after the last chemotherapy cycle. Vitamin B12 (1000 µg) should be given intramuscularly one to three weeks before the first dose of chemotherapy and repeated every nine weeks until four weeks after the last chemotherapy cycle. In addition, methylprednisolone (1 x 32 mg orally or equivalent in whatever formulation is available locally) should be given the day before (day -1 of CT cycle), the day of (day 0), and the day after (day 1) pemetrexed dosing to reduce the risk of severe skin rash.

In case of a delay in chemotherapy administration, atezolizumab administration and/or WT1/DC vaccination may be delayed accordingly, in agreement with the Investigator. A delay in chemotherapy administration due to an AE will not be considered as a protocol deviation.

6.2 Atezolizumab information

6.2.1 Nature of the therapy

Atezolizumab is an Fc-engineered, humanised IgG1 anti-programmed death-ligand 1 (PD-L1) monoclonal antibody produced in Chinese hamster ovary cells by recombinant DNA technology.

6.2.1.1 *Mechanism of action*

Atezolizumab is a humanized IgG1 monoclonal antibody that targets PD-L1 and inhibits the interaction between PD-L1 and its receptors, PD-1 and B7-1 (also known as CD80), both of which function as inhibitory receptors expressed on T cells. Therapeutic blockade of PD-L1 binding by atezolizumab has been shown to enhance the magnitude and quality of tumor-specific T-cell responses, resulting in improved anti-tumor activity[43,44]. Atezolizumab has minimal binding to Fc receptors, thus eliminating detectable Fc-effector function and associated antibody-mediated clearance of activated effector T cells.

6.2.1.2 *Antitumor activity*

Atezolizumab shows anti-tumor activity in both non-clinical models and cancer patients and is being investigated as a potential therapy in a wide variety of malignancies. Atezolizumab is being studied as

a single agent in the advanced cancer and adjuvant therapy settings, as well as in combination with chemotherapy, targeted therapy, and cancer immunotherapy.

Atezolizumab is currently approved by the European Commission for the treatment of urothelial carcinoma, non-small cell lung cancer, small-cell lung cancer, triple-negative breast cancer and hepatocellular carcinoma.

6.2.1.3 *Toxicity in humans*

Atezolizumab has been associated with risks such as the following: infusion-related reactions and immune-mediated hepatitis, pneumonitis, colitis, pancreatitis, type 1 diabetes mellitus, hypothyroidism, hyperthyroidism, adrenal insufficiency, hypophysitis, Guillain-Barré syndrome and demyelinating polyneuropathy, myasthenic syndrome or myasthenia gravis, meningoencephalitis, myocarditis, pericardial disorders, nephritis, myositis, myelitis, facial paresis and severe cutaneous adverse reactions. Immune-mediated reactions may involve any organ system and may lead to hemophagocytic lymphohistiocytosis (HLH) and macrophage activation syndrome (MAS), which are considered to be potential risks for atezolizumab.

Summary of the safety profile

The safety of atezolizumab as monotherapy is based on pooled data in 4739 patients across multiple tumor types. The most common adverse reactions (> 10%) were fatigue (29.9%), decreased appetite (20.0%), rash (20.0%), nausea (19.4%), diarrhea (18.4%), pyrexia (18.3%), cough (18.1%), arthralgia (16.6%), dyspnoea (16.4%), pruritus (13.7%) asthenia (12.9%), back pain (12.5%), vomiting (11.9%), urinary tract infection (11.1%) and headache (10.5%).

The safety of atezolizumab given in combination with other medicinal products, has been evaluated in 4535 patients across multiple tumor types. The most common adverse reactions (≥ 20%) were anemia (36.8%), neutropenia (36.6%), nausea (35.5%), fatigue (33.1%), alopecia (28.1%), rash (27.8%), diarrhoea (27.6%), thrombocytopenia (27.1%), constipation (25.8%), decreased appetite (24.7%) and peripheral neuropathy (24.4%).

Tabulated list of adverse reactions

The Adverse Drug Reactions (ADRs) are listed by MedDRA system organ class (SOC) and categories of frequency in the Table 1 for atezolizumab given as monotherapy or as combination therapy. Adverse reactions known to occur with atezolizumab or chemotherapies given alone may occur during treatment with these medicinal products in combination, even if these reactions were not reported in clinical trials with combination therapy. The following categories of frequency have been used: very common (≥ 1/10), common (≥ 1/100 to < 1/10), uncommon (≥ 1/1,000 to < 1/100), rare (≥ 1/10,000 to < 1/1,000), very rare (< 1/10,000). Within each frequency grouping, adverse reactions are presented in the order of decreasing seriousness.

Table 1. Summary of adverse reactions occurring in patients treated with atezolizumab

**Atezolizumab monotherapy**		**Atezolizumab in combination therapy**
**Infections and infestations**		
Very common	urinary tract infection[a]	lung infection[b]
Common		sepsis[aj]
**Blood and lymphatic system disorders**		
Very common		anaemia, thrombocytopenia[d], neutropenia[e], leukopenia[f]
Common	thrombocytopenia[d]	lymphopenia[g]
Rare	haemophagocytic lymphohistiocytosis	haemophagocytic lymphohistiocytosis
**Immune system disorders**		
Common	infusion-related reaction[h]	infusion-related reaction[h]
**Endocrine disorders**		
Very common		hypothyroidism[i]

Common	hypothyroidism[i], hyperthyroidism[j]	hyperthyroidism[j]
Uncommon	diabetes mellitus[k], adrenal insufficiency[l]	
Rare	hypophysitis[m]	
**Metabolism and nutrition disorders**		
Very common	decreased appetite	decreased appetite
Common	hypokalemia[ae], hyponatremia[af], hyperglycemia	hypokalemia[ae], hyponatremia[af], hypomagnesemia[n]
**Nervous system disorders**		
Very Common	Headache	peripheral neuropathy[o] , headache
Common		syncope, dizziness
Uncommon	Guillain-Barré syndrome[p], meningoencephalitis[q]	
Rare	myasthenic syndrome[r], facial paresis, myelitis	facial paresis
**Eye Disorders**		
Rare	Uveitis	
**Cardiac disorders**		
Rare	myocarditis[s]	
Common	Pericardial disorders[ao]	
Uncommon		Pericardial disorders[ao]
**Vascular disorders**		
Very Common		hypertension[ai]
Common	Hypotension	
**Respiratory, thoracic, and mediastinal disorders**		
Very common	cough, dyspnea	dyspnea, cough, nasopharyngitis[am]
Common	pneumonitis[t], hypoxia[ag],, nasopharyngitis[am]	dysphonia
**Gastrointestinal disorders**		
Very common	nausea, vomiting, diarrhea[u]	nausea, diarrhea[u], constipation, vomiting
Common	abdominal pain, colitis[v], dysphagia, oropharyngeal pain[w], dry mouth	stomatitis, dysgeusia
Uncommon	pancreatitis[x]	
**Hepatobiliary disorders**		
Common	AST increased, ALT increased, hepatitis[y]	AST increased, ALT increased
**Skin and subcutaneous tissue disorders**		
Very Common	rash[z], pruritus	rash[z], pruritus, alopecia[ah]
Common	dry skin	
Uncommon	psoriasis, severe cutaneous adverse reactions[ak]	psoriasis, severe cutaneous adverse reactions[ak]

Rare	Pemphigoid	pemphigoid
**Musculoskeletal and connective tissue disorders**		
Very common	arthralgia, back pain	arthralgia, musculoskeletal pain[aa], back pain
Common	musculoskeletal pain[aa]	
Uncommon	myositis[ab]	
**Renal and urinary disorders**		
Common	blood creatinine increased[c]	proteinuria[ac], blood creatinine increased[c]
Uncommon	nephritis[ad]	
**General disorders and administration site conditions**		
Very Common	pyrexia, fatigue, asthenia	pyrexia, fatigue, asthenia, oedema peripheral
Common	influenza like illness, chills	
**Investigations**		
Common		blood alkaline phosphatase increased

[a] Includes reports of urinary tract infection, cystitis, pyelonephritis, escherichia urinary tract infection, urinary tract infection bacterial, kidney infection, pyelonephritis acute, pyelonephritis chronic, pyelitis, renal abscess, streptococcal urinary tract infection, urethritis, urinary tract infection fungal, urinary tract infection pseudomonal.
[b] Includes reports of pneumonia, bronchitis, lung infection, lower respiratory tract infection, infectious pleural effusion, tracheobronchitis, atypical pneumonia, lung abscess, paracancerous pneumonia, pyopneumothorax, pleural infection, post procedural pneumonia.
[c] Includes reports of blood creatinine increased, hypercreatininaemia.
[d] Includes reports of thrombocytopenia, platelet count decreased.
[e] Includes reports of neutropenia, neutrophil count decreased, febrile neutropenia, neutropenic sepsis, granulocytopenia.
[f] Includes reports of white blood cell count decreased, leukopenia.
[g] Includes reports of lymphopenia, lymphocyte count decreased.
[h] Includes reports of infusion related reaction, cytokine release syndrome, hypersensitivity, anaphylaxis.
[i] Includes reports of anti-thyroid antibody positive, autoimmune hypothyroidism, autoimmune thyroiditis, blood thyroid stimulating hormone abnormal, blood thyroid stimulating hormone decreased, blood thyroid stimulating hormone increased, euthyroid sick syndrome, goitre, hypothyroidism, immune-related hypothyroidism, myxoedema, myxoedema coma, primary hypothyroidism, thyroid disorder, thyroid hormones decreased, thyroid function test abnormal, thyroiditis, thyroiditis acute, thyroxine decreased, thyroxine free decreased, thyroxine free increased, thyroxine increased, tri-iodothyronine decreased, tri-iodothyronine free abnormal, tri-iodothyronine free decreased, tri-iodothyronine free increased, silent thyroiditis, thyroiditis chronic.
[j] Includes reports of hyperthyroidism, Basedow's disease, endocrine ophthalmopathy, exophthalmos.
[k] Includes reports of diabetes mellitus, type 1 diabetes mellitus, diabetic ketoacidosis, ketoacidosis.
[l] Includes reports of adrenal insufficiency, blood corticotropin decreased, glucocorticoid deficiency, primary adrenal insufficiency, secondary adrenocortical insufficiency.
[m] Includes reports of hypophysitis, temperature regulation disorder.
[n] Includes reports of hypomagnesaemia, blood magnesium decreased.
[o] Includes reports of neuropathy peripheral, autoimmune neuropathy, peripheral sensory neuropathy, polyneuropathy, herpes zoster, peripheral motor neuropathy, neuralgic amyotrophy, peripheral sensorimotor neuropathy, toxic neuropathy, axonal neuropathy, lumbosacral plexopathy, neuropathic arthropathy, peripheral nerve infection, neuritis, immune-mediated neuropathy.
[p] Includes reports of Guillain-Barré syndrome, demyelinating polyneuropathy.
[q] Includes reports of encephalitis, encephalitis autoimmune, meningitis, photophobia.
[r] Includes reports of myasthenia gravis.
[s] Includes reports of autoimmune myocarditis, autoimmune myocarditis, and immune-mediated myocarditis.
[t] Includes reports of pneumonitis, lung infiltration, bronchiolitis, immune-related pneumonitis, interstitial lung disease, alveolitis, lung opacity, pulmonary toxicity, radiation pneumonitis.
[u] Includes reports of diarrhea, defecation urgency, frequent bowel movements, diarrhea hemorrhagic, gastrointestinal hypermotility.
[v] Includes reports of colitis, autoimmune colitis, colitis ischaemic, colitis microscopic, colitis ulcerative, diversion colitis, immune-related enterocolitis.
[w] Includes reports of oropharyngeal pain, oropharyngeal discomfort, throat irritation.
[x] Includes reports of autoimmune pancreatitis, pancreatitis, pancreatitis acute, lipase increased, amylase increased.
[y] Includes reports of ascites, autoimmune hepatitis, hepatocellular injury, hepatitis, hepatitis acute, hepatitis toxic, hepatotoxicity, liver disorder, drug-induced liver injury, hepatic failure, hepatic steatosis, hepatic lesion, esophageal varices hemorrhage, varices esophageal.
[z] Includes reports of acne, acne pustular, blister, blood blister, dermatitis, dermatitis acneiform, dermatitis allergic, dermatitis exfoliative, drug eruption, eczema, eczema infected, erythema, erythema of eyelid, eyelid rash, fixed eruption, folliculitis, furuncle, hand dermatitis, lip blister, oral blood blister, palmar-plantar erythrodysaesthesia syndrome, pemphigoid, rash, rash erythematous, rash follicular, rash generalised, rash macular, rash maculo-papular, rash papular, rash papulosquamous, rash pruritic, rash pustular, rash vesicular, scrotal dermatitis, seborrhoeic dermatitis, skin exfoliation, skin toxicity, skin ulcer.
[aa] Includes reports of musculoskeletal pain, myalgia, bone pain.
[ab] Includes reports of myositis, rhabdomyolysis, polymyalgia rheumatica, dermatomyositis, muscle abscess, myoglobin urine present.
[ac] Includes reports of proteinuria, protein urine present, haemoglobinurea, urine abnormality,nephrotic syndrome, albuminuria.

[ad] Includes reports of nephritis, autoimmune nephritis, Henoch-Schonlein Purpura nephritis, paraneoplastic glomerulonephritis, tubulointerstitial nephritis.
[ae] Includes reports of hypokalemia, blood potassium decreased.
[af] Includes reports of hyponatremia, blood sodium decreased.
[ag] Includes reports of hypoxia, oxygen saturation decreased, pO2 decreased.
[ah] Includes reports of alopecia, madarosis, alopecia areata, alopecia totalis, hypotrichosis.
[ai] Includes reports of hypertension, blood pressure increased, hypertensive crisis, blood pressure systolic increased, diastolic hypertension, blood pressure inadequately controlled, retinopathy hypertensive, hypertensive nephropathy, essential hypertension, orthostatic hypertension.
[aj] Includes reports of sepsis, septic shock, urosepsis, neutropenic sepsis, pulmonary sepsis, bacterial sepsis, klebsiella sepsis, abdominal sepsis, candida sepsis, escherichia sepsis, pseudomonal sepsis, staphylococcal sepsis.
[ak] Includes reports of dermatitis bullous, exfoliative rash, erythema multiforme, dermatitis exfoliative generalised, toxic skin eruption, Stevens-Johnson syndrome, drug reaction with eosinophilia and systemic symptoms, toxic epidermal necrolysis, cutaneous vasculitis.
[al] Includes reports of cystitis noninfective and immune-mediated cystitis.
[am] Includes reports of nasopharyngitis, nasal congestion and rhinorrhoea.
[an] Includes reports of psoriasis, dermatitis psoriasiform, guttate psoriasis.
[ao] Includes reports of pericarditis, pericardial effusion, cardiac tamponade and pericarditis constrictive.

6.2.2 Pharmaceutical data

6.2.2.1 *Formulation*

Atezolizumab 1200 mg and atezolizumab 840 mg will be supplied by Roche as a sterile liquid in single-use, glass vials with a butyl rubber stopper and an aluminum seal with a plastic, grey flip-off cap, containing respectively 20 mL (1200 mg) or 14mL (840 mg) of atezolizumab concentrate for solution for infusion.

6.2.2.2 *Manufacturer*

Name and address of the manufacturers of the biological active substance:

F. Hoffmann-La Roche AG
Grenzacherstrasse 124
4070 Basel
SWITZERLAND

and

Roche Diagnostics GmbH
Nonnenwald 2
82377 Penzberg x
GERMANY

Name and address of the manufacturer responsible for batch release:

Roche Pharma AG
Emil-Barell-Strasse 1
79639 Grenzach-Whylen
GERMANY

6.2.2.3 *Atezolizumab preparation*

For intravenous administration, atezolizumab 1200 mg (20mL vial) and atezolizumab 840 mg (14mL vial) will be administered in IV infusion bags containing 0.9% sodium chloride (NaCl) and infusion lines equipped with 0.2 or 0.22 μm in-line filters. The IV bag may be constructed of polyvinyl chloride, polyolefin, polyethylene, or polypropylene, the IV infusion line may be constructed of polyvinyl chloride, polyethylene, or polybutadiene and the 0.2 or 0.22 μm in-line filter may be constructed of polyethersulfone or polysulfone. The use of administration supplies composed of materials other than those listed should be avoided if possible.

Atezolizumab can be diluted to concentrations between 3.2 mg/mL and 16.8 mg/mL in IV bags containing 0.9% NaCl. Atezolizumab must be prepared/diluted under appropriate aseptic conditions as it does not contain antimicrobial preservatives. The prepared solution for infusion should be used immediately to limit microbial growth in case of potential accidental contamination. If not used

immediately, in-use storage time and conditions prior to use are the responsibility of the user. For flat or fixed dosing (e.g. 1200 mg or 1680 mg) in IV infusion bags, the dose solution may be stored at 2°C–8°C for 24 hours or at ambient temperature ≤ 25°C for 8 hours. This time includes storage and time for administration for infusion. If the dose solution is stored at 2°C–8°C, it should be removed from refrigeration and allowed to reach room temperature prior to administration. Do not shake or freeze infusion bags containing the dose solution.

6.2.2.4 *Atezolizumab administration*

Atezolizumab (1200 mg) will be administered on day 0 of each 3-weekly platinum/pemetrexed-based chemotherapy cycle. Atezolizumab should be administered before chemotherapy administration as an IV infusion over 60 (±15) minutes. If the first infusion is tolerated, all subsequent infusions may be delivered over 30 (±10) minutes. Additional atezolizumab treatment (1680 mg) after the study treatment schedule can be administered (optional) at 4-weekly intervals (± 1 week) as an IV infusion over 30-60 minutes.

First infusion

- No premedication is allowed.
- Record patient's vital signs (heart rate, respiratory rate, blood pressure, and temperature) within 60 minutes before starting infusion.
- Infuse atezolizumab (1200 mg in a 250 mL 0.9% NaCl IV infusion bag) over 60 (±15) minutes
- If clinically indicated, record patient's vital signs (heart rate, respiratory rate, blood pressure, and temperature) during the infusion at 15, 30, 45, and 60 minutes (±5-minute windows are allowed for all timepoints).
- If clinically indicated, record patient's vital signs (heart rate, respiratory rate, blood pressure, temperature) at 30 (±10) minutes after the infusion.
- Patients will be informed about the possibility of delayed post-infusion symptoms and instructed to contact their study physician if they develop such symptoms.

Subsequent infusions:

- If the patient experienced infusion-related reaction during any previous infusion, premedication with antihistamines, anti-pyretics, and/or analgesics may be administered for Cycles ≥2 at the discretion of the treating physician.
- Record patient's vital signs (heart rate, respiratory rate, blood pressure, and temperature) within 60 minutes before starting infusion.
- If the patient tolerated the first infusion well without infusion-associated adverse events, the second infusion may be delivered over 30 (±10 minutes) minutes.
- If no reaction occurs, continue subsequent infusions over 30 (±10 minutes).
- Continue to record vital signs within 60 minutes before starting infusion, and during and after the infusion if clinically indicated.
- If the patient had an infusion-related reaction during the previous infusion, the subsequent infusion must be delivered over 60 (±15) minutes.
- Record patient's vital signs (heart rate, respiratory rate, blood pressure, and temperature) during the infusion if clinically indicated or patient experienced symptoms during the previous infusion.
- Record patient's vital signs (heart rate, respiratory rate, blood pressure, and temperature) 30 min (±10) after the infusion, if clinically indicated or patient experienced symptoms during previous infusion.

6.2.2.5 *Storage*

Atezolizumab must be refrigerated at 2°C−8°C upon receipt until use. Atezolizumab and the diluent vials should not be used beyond the expiration date provided by the manufacturer. No preservative is used, therefore the vial is intended for single use only. Discard any unused portion of drug remaining in a vial. Vial contents should not be frozen or shaken and should be protected from light by keeping the vial in the outer carton.

6.2.2.6 *Disposal*

Any unused medicinal product or waste material should be disposed of in accordance with local requirements.

6.2.3 Dose modification, delay or discontinuation

Dose modifications

Dose modifications to atezolizumab are not permitted.

Treatment interruption or discontinuation

Intolerance results in either a delay or omission of an atezolizumab administration or discontinuation of atezolizumab therapy. Atezolizumab treatment may be temporarily suspended in patients experiencing toxicity considered to be related to study treatment. If corticosteroids are initiated for treatment of the toxicity, they must be tapered over ≥ 1 month to ≤ 10 mg/day oral prednisone or equivalent before atezolizumab can be resumed. If atezolizumab is withheld for > 105 days after event onset, the patient will be discontinued from atezolizumab.
In case of a delay in atezolizumab administration, the WT1/DC vaccine may be delayed accordingly, in agreement with the Investigator. Upon prolonged delay of atezolizumab administration, WT1/DC vaccination can be restarted when the Investigator and treating physician judge that the participant's clinical situation justifies additional administrations. Delays in atezolizumab administration and/or WT1/DC vaccination due to an AE are not considered as a protocol deviation.

If atezolizumab therapy is discontinued, WT1/DC vaccination can be restarted when the Investigator and treating physician judge that the participant's clinical situation justifies additional administrations.

6.3 Vaccine information

6.3.1 Nature of the therapy

Ex vivo generated autologous *WT1-DC-LAMP* mRNA-electroporated monocyte-derived DCs suspended in sterile saline solution with 5% human albumin.

6.3.1.1 *Mechanism of action*

Immunotherapy (biological response modifier therapy): stimulation of effective anti-mesothelioma cellular immunity.

6.3.1.2 *Experimental antitumor activity*

Human studies have demonstrated *in vitro* and *in vivo* capacity of WT1-loaded mature DCs to activate WT1-specific T cells and natural killer (NK) cells.

6.3.1.3 *Toxicity in humans*

WT1 is classified as a tumor-associated antigen, meaning that it is expressed in tumor tissues as well as in normal, healthy tissues. In humans, WT1 is expressed in various normal tissues, including gonads, kidney and hematopoietic cells. However, based on the toxicity data compiled from 21 phase I and II clinical trials of WT1-targeted immunotherapy in cancer patients (n=158), the risk of autoimmunity associated with targeting of WT1 appears to be low.[45] Severe immune-related adverse events (grade 3 or 4 leukopenia [n=2] and thrombocytopenia [n=1], graded according to the National Cancer Institute's Common Toxicity Criteria (NCI CTCAE) due to expression of WT1 in hematopoietic tissue have only been reported in 3 patients with ongoing hematological disease (myelodysplasia, AML).[45] Autoimmune reactions towards normal non-hematopoietic, WT1-expressing tissues have not been observed so far.[45]

DC-based immunotherapy has been introduced in the clinic in 1996.[12] Since then, different types of DC vaccines have been administered to thousands of patients.[46] From this experience, it can be concluded

that DC vaccination is generally safe and well-tolerated. Among the most commonly reported side effects are local injection site reactions (erythema, itching) and flu-like symptoms (fever, myalgia), but these are generally mild (NCI CTCAE grade 1 or 2) and transient. For WT1/DC vaccination particularly, mild IMP-related reactions at the injection site (i.e. swelling, redness, itch) have been reported regularly, as well as isolated cases of other mild adverse reactions following WT1/DC vaccination (refer to section 6.3.3). No serious adverse events (SAEs) possibly, probably or definitely related to the vaccine developed during these trials, confirming its overall beneficial safety profile.

6.3.2 Pharmaceutical data

6.3.2.1 *Formulation*

WT1/DC vaccines for administration will be supplied as a sterile, pyrogen-free, cell solution in saline with 5% human albumin. Each vaccine will contain 8-10 x 10^6 *WT1-DC-LAMP* mRNA-electroporated DCs in 500 µL saline solution with 5% human albumin.

6.3.2.2 *Manufacturer*

Cryopreserved WT1/DC vaccines will be produced and manufactured in compliance with GMP standards and all other applicable regulations and requirements, including those prescribed by the Belgian Federal Agency for Medicines and Health Products (FAMHP).

For other details, see also the latest version of the IMPD.

anicells
Galileilaan 21, B-2845 Niel, Belgium
[redacted]

6.3.2.3 *Vaccine preparation*

WT1/DC vaccine preparation for administration according to the latest version of the reconstitution handling manual will be performed at the CCRG facility under responsibility of the hospital pharmacy as agreed in the service level agreement.

For other details, see also the latest version of the reconstitution handling manual.

Center for Cell Therapy and Regenerative Medicine (CCRG)
Antwerp University Hospital (UZA)
Drie Eikenstraat 655, B-2650 Edegem, Belgium
[redacted]

7.1.1.1 *Vaccine administration*

WT1/DC vaccines will be administered at the Division of Hematology of the Antwerp University Hospital.

For other details, see also the latest version of the reconstitution handling manual.

Division of Hematology
Antwerp University Hospital (UZA)
Drie Eikenstraat 655, B-2650 Edegem, Belgium
[redacted]

8.1.1.1 *Storage*

After *ex vivo* production, the WT1/DC vaccines are cryopreserved in aliquots in pretested human AB serum supplemented with 2% (w/v) glucose and 10% DMSO, at temperatures below -130°C.

8.1.1.2 *Stability*

Vaccine stability is guaranteed for up to 3,5 years when stored at temperatures below -130°C (*for more details see the latest version of the IMPD and the reconstitution handling manual)*. The vaccine needs to be administered as soon as possible, within 3 hours following the end of the preparation procedure after thawing of the cryopreserved WT1/DCs.

8.1.1.3 *Packaging, storage and dispensing*

Packaging and labeling of the cryopreserved WT1/DC vaccines will be in accordance with GMP for clinical trials and the Quality Manual of the GMP manufacturing facility, where records will be maintained. It will also keep an inventory of all vaccines produced per included subject, including all relevant information related to the production thereof. The inventory must be available for monitoring, auditing or inspection.
For more details, see also the latest version of the IMPD.

For dispensing of the thawed WT1/DC vaccine, we refer to 6.3.2.1. '*Formulation*' and 5.2.3.2 and 6.3.2.3. '*Vaccine preparation*'.

8.1.1.4 *Drug destruction procedures*

All unused WT1/DC vaccines will be stored for use in the context of validation procedures and/or scientific research, in accordance with the signed informed consent. Otherwise, unused WT1/DC vaccines will be destroyed after end of study. A certificate of destruction will be completed by CCRG and approved by the sponsor.

8.1.2 Vaccine dose omission or discontinuation

Dose modifications for the WT1/DC vaccine are not foreseen within the current study protocol, resulting in either omission of a vaccine dose or discontinuation of WT1/DC vaccination in case of intolerance.

The principal WT1/DC vaccine-related toxicities seen in previous studies are:

- Local injection site reactions (common): erythema, pruritus, induration, pain, rash, and rarely crusts or vesicles.
  Reactions are generally mild or moderate (NCI CTCAE Grade 1 or 2), but if the patient feels reactions interfere with activities of daily living (ADL), the next WT1/DC vaccine may be omitted if there is residual toxicity interfering with ADL at that time.
- Subfebrility/fever (uncommon)

Furthermore, isolated cases of following adverse reactions following WT1/DC vaccination were reported in previous studies (very uncommon):

- Flare-up of psoriasis (mild)
- Malaise and fatigue (mild)
- Skin reaction (redness, itch and/or stinging sensation) on the contralateral side of injection (arm area and/or neck and chest area) (mild)
- Rash in neck and upper arms (moderate)
- Nausea (mild)
- Increased sensitivity to a thoracotomy and biopsy related scar (mild)

Possible toxicity not seen in previous studies includes:

- Hypersensitivity/allergic reactions
  In case of NCI CTCAE Grade 2 hypersensitivity/allergic reactions, vaccination must be discontinued for that patient except when the Investigator, in agreement with the Coordinating investigator, judges that it is in the best interest of the patient to continue. In case of Grade 3 hypersensitivity/allergic reactions that cannot be controlled by corticosteroids or any Grade 4 hypersensitivity/allergic reactions, vaccination must be discontinued permanently for that patient.
- Any other significant (NCI CTCAE Grade 3 or 4) toxicity.

If deemed reasonably likely that the AE may be associated with administration of the WT1/DC vaccine, vaccination for that patient must be omitted until toxicity ≤ Grade 1. If significant toxicity returns or is irreversible, vaccination must be discontinued permanently.

9 Response evaluation

9.1 Definition of evaluable patients

Except for feasibility assessment, analyses will be performed on all evaluable patients. Patients are considered evaluable if they completed at least the first cycle of the study treatment scheme.

9.2 Analyses of responses

9.2.1 Feasibility and safety (Primary objective)

Safety will be assessed in all evaluable patients by reporting:

- Local toxicity (e.g. skin reactions at injection site) (for WT1/DC vaccination only)
- Systemic toxicity, according to the latest version of the NCI CTCAE and Common Toxicity Criteria (CTC). *For more details, see: http://ctep.cancer.gov/protocolDevelopment/electronic_applications/ctc.htm.*

Endpoint for safety of the combination of first-line platinum/pemetrexed-based chemotherapy in combination with atezolizumab and WT1/DC vaccination is the occurrence of AEs and SAEs during investigational treatment administration and during follow-up:

- Proportions of patients that experienced (S)AEs possibly, probably or definitely related to pemetrexed and/or cisplatin/carboplatin and/or atezolizumab and/or WT1/DC vaccination
- Number and grade of AEs and SAEs

From a safety perspective, the study will be considered successful when ≤ 40% of patients experience immunotherapy-related (i.e. atezolizumab- or WT1/DC vaccine-related) SAEs within 6 months after initiating therapy.

Feasibility will be assessed based on the success of the completion of the study treatment scheme and will be evaluated for all patients enrolled in the study (both evaluable and non-evaluable).

Endpoint for feasibility is the proportion of patients who completed study treatment schedule (i.e. administration of four platinum/pemetrexed-based chemotherapy cycles (CT_{1-4}) in combination with four atezolizumab treatments (A_{1-4}) and four WT1/DC vaccinations (V_{1-4}).

From a feasibility perspective, the study will be considered successful when >66% of patients complete the study treatment schedule.

9.2.2 Clinical efficacy (Secondary and exploratory objectives)

Patients will be followed for clinical responses, disease progression and survival. Tumor evolution assessment will be performed according to the latest modified Response Evaluation Criteria In Solid Tumors (mRECIST) for malignant pleural mesothelioma and RECIST 1.1 criteria for metastatic lesions (http://www.recist.com/). Clinical response analysis will be performed on all evaluable patients.

Endpoints for clinical efficacy of first-line platinum/pemetrexed-based chemotherapy in combination with atezolizumab and WT1/DC vaccination (secondary objective) include:

- Best overall response (BOR), duration of response (DOR), disease control rate (DCR), objective response rate (ORR) and progression free survival (PFS)
- Overall survival (OS) from diagnosis and start of first-line treatment

Clinical efficacy of next-line treatments in epithelioid MPM patients following or in combination with WT1/DC vaccination may be determined (exploratory objective). Possible outcomes that may be considered are BOR, DOR, DCR, ORR, PFS and OS.

Definitions for parameters determining clinical response:

- BOR will be determined per patient as the best overall response designation. The BOR categories are complete response (CR), partial response (PR), stable disease (SD) and progressive disease (PD).
- DOR will be calculated for patients with an objective response as the time between the first date of the first documented tumor response (CR or PR) and the subsequent date of the objectively documented disease progression or death, whichever occurs first, or the last tumor assessment in case of censoring.
- DCR is defined as the proportion of patients whose BOR is either CR, PR or SD, where the dominator is the total number of evaluable patients.
- ORR is defined as the proportion of patients whose BOR is either CR or PR, where the dominator is the total number of evaluable patients.
- PFS is defined as the time (in months) between start of the platinum/pemetrexed-based chemotherapy treatment and the date of progression or death due to any cause, whichever occurs first. At the time of analysis, patients without a recorded event will be censored at the time of the last objective disease assessment.
- OS is defined as the time (in months) between diagnosis/start of treatment and death due to any cause. At the time of analysis, patients without a recorded event will be censored at the time they were last known to be alive.

Follow-up after the end of study (*see section 5.2.4.3*) can be done according to local practice.

9.2.3 **Immunological responses and biomarker analysis** (Secondary and exploratory objectives)

Systemic (secondary objective) and tumor biopsy (exploratory) immunological response analysis will be performed to determine the baseline immunological status and the immunogenicity of the proposed chemo-immunotherapy regimen by evaluating the development of effective anti-mesothelioma immunity as described in section 5.2.4.4 and 5.2.4.5 (secondary objective).

The immunogenicity endpoints include, but are not limited to, the following measures of (anti-tumor) immune responses:

- Functional WT1-specific T cell responses (secondary)

Biomarkers can be identified based on associations with clinical and immunological responses following chemo-immunotherapy (exploratory).

10 Safety evaluation

The Investigator and the site staff are responsible for the detection and documentation of events meeting the criteria and definition of an AE or SAE as provided in this protocol.

Each patient will be instructed to contact the Investigator immediately after she/he manifests any sign or symptom they perceive as serious.

10.1 Definitions

10.1.1 Adverse event

An AE is any new undesirable medical experience or change of an existing condition that occurs during or after administration of an investigational agent, whether or not it is considered agent-related.

Abnormal laboratory findings considered by the Investigator to be clinically significant, for example, those that are unusual or unusually severe for the population being studied, will also be considered adverse events.

10.1.2 Serious Adverse event

A SAE is any experience that suggests a significant hazard to the patient and includes any event that:

- is fatal
- is life-threatening (places the patient at immediate risk of death)
- requires or prolongs hospitalization
- is permanently disabling
- is a congenital abnormality/birth defect, or
- requires medical/surgical intervention to prevent any of the above

10.1.3 Adverse events of special interest

Adverse events of special interest (AESI) for this study are as follows:

- Cases of potential drug-induced liver injury that include an elevated ALT or AST in combination with either an elevated bilirubin or clinical jaundice, as defined by Hy's Law
- Suspected transmission of an infectious agent by a study treatment, as defined below:
  Any organism, virus, or infectious particle (e.g., prion protein transmitting transmissible spongiform encephalopathy), pathogenic or non-pathogenic, is considered an infectious agent. A transmission of an infectious agent may be suspected from clinical symptoms or laboratory findings that indicate an infection in a patient exposed to a medicinal product. This term applies only when a contamination of the study treatment is suspected.
- Systemic lupus erythematosus
- Events suggestive of hypersensitivity, infusion-related reactions, cytokine-release syndrome, HLH, and MAS
- Nephritis
- Ocular toxicities (e.g., uveitis, retinitis, optic neuritis)
- Grade ≥ 2 cardiac disorders (e.g., atrial fibrillation, myocarditis, pericarditis)
- Vasculitis
- Autoimmune hemolytic anemia
- Severe cutaneous reactions (e.g., Stevens-Johnson syndrome, dermatitis bullous, toxic epidermal necrolysis)

10.2 Severity of adverse events

The severity of adverse events should be assessed according to the latest version of the National Cancer Institute CTC Scale. The following definitions should be used for adverse events that are not specified in the CTC scale:

- Mild (Grade 1)

The AE is mild; asymptomatic or mild symptoms; clinical or diagnostic observations only; intervention not indicated.

- Moderate (Grade 2)

  The AE is moderate; minimal, local or noninvasive intervention indicated; limiting age-appropriate instrumental activities of daily living (ADL). Instrumental ADL refer to preparing meals, shopping for groceries or clothes, using the telephone, managing money, etc.
- Severe (Grade 3)

  The AE is severe or medically significant but not immediately life-threatening; hospitalization or prolongation of hospitalization indicated; disabling; limiting self care ADL. Self care ADL refer to bathing, dressing and undressing, feeding self, using the toilet, taking medications, and not bedridden.
- Life-threatening (Grade 4)

  The AE is life-threatening; urgent intervention indicated.
- Death (Grade 5)

  Death related to AE.

10.3 Relationship of adverse events to study agents

The relationship of an AE to the investigational agents will be determined by the Investigator as either related or non-related, based on their clinical judgment. The following criteria should be used as guidance to assess the AE as related or non-related:

- Definitely related

  An AE that occurs at a reasonable time interval after administration of the investigational agent, that follows a known response pattern to the investigational agent, that improves after stopping the investigational agent and that reappears after repeated exposure to the investigational agent (rechallenge). Depending on the nature of the adverse event, rechallenge may not be possible, in which case only a probable relationship can be established.
- Probably related

  An AE that occurs at a reasonable time interval after administration of the investigational agent, that follows a known response pattern to the investigational agent, that improves after stopping the investigational agent, and that cannot be reasonably explained by the known characteristics of the patient's clinical state or by other therapies.
- Possibly related

  An AE that occurs at a reasonable time interval after administration of the investigational agent, that follows a known response pattern to the investigational agent, but that could have been produced by the patient's clinical state or by other therapies.
- Unlikely related

  An AE for which the temporal sequence is unlikely to have had any reasonable association with the event and/or the event could have been produced by the patient's clinical state or other concomitant therapy.
- Not related

  An AE for which sufficient information exists to indicate that the etiology is unrelated to the investigational agent. Another etiology must be specified

There may be situations when an SAE has occurred and the Investigator has minimal information to include in the initial report to the Sponsor. However, it is important that the Investigator always assesses the causality for every event prior to submission of the SAE form to the Sponsor. The Investigator may change his/her opinion of causality in the light of follow-up information, amending the SAE information accordingly. The causality assessment is one of the criteria used when determining regulatory reporting requirements.

10.4 Reporting procedures

- During first-line treatment (including continuation of atezolizumab administration and/or WT1/DC vaccination), all AEs must be recorded on the patient's CRF until 90 days after the final investigational treatment. During next-line treatments with continuation of WT1/DC vaccination, only AEs probably, possibly or definitely related to WT/DC vaccination and/or the combination of WT1/DC vaccination with a next-line anti-mesothelioma treatment must be reported in the patient's CRF until 90 days after final WT1/DC vaccination. The following information should be provided in the patient's CRF: a description of the event, date of onset and resolution, severity, relationship to investigational agent(s), action taken and outcome. Patients experiencing AEs should be followed carefully until the condition resolves, and every effort should be made to clarify the underlying cause.
- During first-line treatment, all AESIs and all SAEs (irrespective of suspected causation), including deaths which occur while the patient is on study or within 90 days of the last day on which an investigational agent was administered, must be reported by written document by the Investigator to the Sponsor of the study within one working day of discovery, using the Serious Adverse Event Form. Death related to progressive disease will not be reported as an SAE. During next-line treatments with continuation of WT1/DC vaccination, all AESIs and all SAEs should be logged in the patient's CRF, but only SAE probably, possibly or definitely related to WT1/DC vaccination and/or the combination of WT1/DC vaccination with a next-line anti-mesothelioma treatment must be reported by written document by the Investigator to the Sponsor of the study within one working day of discovery, using the Serious Adverse Event Form.
- The Investigator of the study who is informed of the SAE will notify by written document the Sponsor of the study if the adverse event is possibly, probably or definitely related to the DC injection. Follow-up information related to SAEs and reportable deaths must be submitted to the Sponsor by phone, fax, or e-mail, as soon as relevant data are available.
- If the trial is discontinued for any reason, the Coordinating investigator will notify the EC and provide the reasons for discontinuation.

10.5 Events or outcomes not qualifying as SAEs

An event which is part of or caused by the natural course of the disease under study (i.e. disease progression, recurrence) is captured as an efficacy measure and does not need to be reported as an SAE.

Note: Death related to progressive disease will not be reported as an SAE.

Note: If the Investigator considers that there was a causal relationship between the study treatment/procedures and disease progression and/or death, then this must be reported as an SAE.

11 Statistical considerations

11.1 Sample size and power calculations

This study is intended to accrue a total number of 15 evaluable patients. In Table 2, the probabilities of concluding success or failure are listed for an acceptable toxicity rate of ≤40% and a study population of 15 patients. For a true underlying toxicity rate of ≤40%, the probability of concluding success is at least 61%, while that of concluding failure is ≤10%.

Table 2. Probability of concluding success and failure as a function of the true underlying toxicity rate

True underlying toxicity rate	Probability of concluding success (≤ 40% toxicity rate)	Probability of concluding failure (>55% toxicity rate)	Precision (half width of 95% CI)
5%	100%	<0.01%	0.15
10%	100%	<0.01%	0.18
15%	100%	<0.01%	0.20
20%	98%	0.1%	0.22
25%	94%	0.4%	0.23
30%	87%	2%	0.24
35%	75%	4%	0.25
40%	61%	10%	0.26
45%	45%	18%	0.26
50%	30%	30%	0.26
55%	18%	45%	0.26
60%	10%	61%	0.26
65%	4%	75%	0.25
70%	2%	87%	0.24
75%	0.4%	94%	0.23
80%	0.1%	98%	0.22
85%	<0.01%	100%	0.20
90%	<0.01%	100%	0.18
95%	<0.01%	100%	0.15

11.2 Statistical analyses

11.2.1 Patient characteristics and baseline comparisons

Demographic and other baseline characteristics (e.g., age, sex, smoking status, asbestos exposure, tumor stage, WHO performance score, treatment duration, follow-up period) will be summarized. For categorical variables, frequencies and percentages will be reported. Where values are missing, percentages will be calculated for the available cases, and the denominator will be mentioned. Continuous variables will be summarized as mean with standard deviation (or median and interquartile range as appropriate), and range (min-max).

11.2.2 Analysis of primary objectives

Feasibility will be assessed based on the definition in section 7.2.1 and reported as percentage with 95% Clopper-Pearson confidence interval (CI). The amount of produced and administered WT1/DC vaccines will be summarized as mean with standard deviation, and range (min-max).

For safety, frequency and percentages with 95% Clopper-Pearson CI of patients who experienced (S)AEs as defined in section 7.2.1 will be reported. Number and grade of (S)AEs will be summarized.

11.2.3 Analysis of secondary objectives

For the survival measures (PFS and OS; see section 7.2.2) median times (or lower bound) and Kaplan-Meier curves will be determined at the different analysis points (see section 9.3.2). Median survival time with 95% CI will be determined. These survival measures will be updated after end of study at the Investigator's discretion.

BOR and DOR will be determined for each patient after first-line chemo-immunotherapy and summarized. Proportions (i.e. ORR and DCR) will be reported with corresponding 95% Clopper-Pearson CI. See section 7.2.2 for the different definitions.

Systemic immunogenicity (see section 7.2.3) will be reported with summary measures (mean, standard deviation or median and interquartile range as appropriate, and range (min-max)). The difference between these investigated parameters at each immunomonitoring blood sample collection time point will be compared and the association with indicators of clinical activity and survival will be studied.

11.2.4 Analysis of exploratory objectives

Clinical efficacy of next-line treatments in epithelioid MPM patients following or in combination with WT1/DC vaccination may be determined. BOR and DOR may be determined and summarized and ORR and DCR proportions may be reported with corresponding 95% CI. See section 7.2.2 for the different definitions. In addition, PFS and OS after next-line treatments following or in combination with WT1/DC vaccination, median times (or lower bound) and Kaplan-Meier curves may be determined. Median survival time with 95% CI may be determined.

For biomarker identification, associations between the investigated parameters as well as with clinical and immunological responses following chemo-immunotherapy and survival can be studied graphically and if homogeneity of population allows association measures can be calculated. For quality of life evaluation, all scores can be calculated according to the manuals. Summary scores can be calculated at each assessment point using descriptive statistics. Summary measures can be calculated and a plot of quality of life scores over time can be considered. Associations with indicators of clinical activity can be studied graphically and associations measures can be calculated.

11.3 Analyses and reporting

A first analysis will be done when 8 patients have survived 24 months after diagnosis (i.e. day of tumor biopsy). All available data, feasibility, safety, immunogenicity and indicators of clinical efficacy will be evaluated at this time.

A second analysis will be done when all patients have reached an event (death) or 24 months after diagnosis (i.e. day of tumor biopsy). All available data, feasibility, safety, immunogenicity and indicators of clinical efficacy will be reevaluated at this time.

At end of study (i.e. 90 days after final WT1/DC vaccination and/or atezolizumab administration or 24 months after diagnosis, whichever occurs later), final analysis for safety, immunogenicity and indicators of clinical efficacy will be done. If the time between the second and final analysis is less than 12 months, only final analysis will be performed.

After end of study, the Sponsor has the right to collect data on the patient's mesothelioma-related disease status (including imaging data) and anti-mesothelioma treatments, as well as to collect survival data as stated in the informed consent form.

For the survival measures we are interested in calculating median survival with the corresponding confidence interval. For this we need the 50th and 45th percentile of the survival function. At each

analysis time we will evaluate if these percentiles are reached and if so we will calculate these measures. Before this time it will only be possible to calculate a lower bound on the median survival.

At each analysis time point, percentages and descriptive statistics will be reported and compared to published data in a comparable patient population. Inference for the survival measures will be calculated at the earliest analysis time possible if conditions are met (survival function has reached 45th percentile).

Any extra post-hoc exploratory analyses performed to provide support for planned analyses, but not mentioned in the SAP will be documented and reported in appendices and clearly marked as unplanned analyses in any publication.

12 Administrative matters

12.1 Study documentation

12.1.1 Investigator records

Each Investigator must keep adequate and accurate patient records to enable the appropriate and required documentation of the study and subsequent verification of the collected data. In addition, the current International Conference on Harmonisation of Technical Requirements for Registration of Pharmaceuticals for Human Use (ICH) - Harmonised Tripartite Guidelines for Good Clinical Practice (GCP) require that Investigators maintain all source information used to compile the patient's records to enable confirmation of the data collected on the electronic case report forms (eCRFs). Investigators must keep all original source information and pertinent study documentation (e.g., signed informed consents) for at least 30 years after completion or discontinuation of the study. After that period documents may be destroyed, subject to local regulations.

12.1.2 Electronic case report forms

eCRFs will be supplied by the Sponsor for recording all study data. It is the responsibility of the Principal investigator to ensure that study data are recorded in an accurate, adequate and timely manner.

eCRFs must be completed for each patient enrolled in this study, including patients who did not start with the investigational treatment. When a patient completes a visit, it is anticipated that relevant sections of the eCRF will be completed by the Principal investigator (or designated staff as documented on the Delegation Log) as soon as possible after the last data becoming available. Similarly, when a patient completes the study, it is anticipated that all relevant eCRF pages will be completed promptly after the last data become available. If a patient withdraws from the study, the reason must be noted on the eCRF. As soon as the patient has completed/withdrawn from the study and the eCRF is completed, the Principal investigator will sign the study conclusion pages of the eCRF to confirm that they have reviewed the data and that the data are complete and accurate.

eCRFs are reviewed at the study site by a monitor on behalf of the Sponsor. Errors detected by eCRF review may necessitate clarification or correction of errors with documentation and approval by the Principal investigator. In all cases, the Principal investigator remains accountable for the study data.

At the study conclusion, the Sponsor will archive the study data in accordance with internal procedures.

Specific instructions for use of the eCRF will be included in the eCRF guidelines.

12.2 Monitoring procedures

Data monitoring will be performed by monitors of the Clinical Trial Center (CTC) of the UZA to check the completeness of patients' records, to ensure that all aspects of the protocol are followed and to verify compliance with GCP guidelines.

During a training session, the monitors will be trained by the study coordinators for the protocol, eCRF and other study documents. During the study, the monitors will visit the sites regularly in accordance with the study-specific monitoring plan, which will be approved by the Principal investigator. Monitoring visits include, but are not restricted to, verification of the presence of a valid ICF, adherence to the inclusion/exclusion criteria, documentation of SAEs, and adequacy of data that will be used for all

primary variables. Additional checks of the consistency of the source data with the eCRFs are performed according to the study-specific monitoring plan. Key study personnel must be available to assist the field monitor during these visits.

The Principal investigator must give the study monitor access to relevant hospital or clinical records to confirm their consistency with the eCRF entries. All information on eCRF must be traceable to these source documents in the patient's file. No information of these records about the identity of the patients will leave the study center. The Investigators must also keep the original ICF signed by the patient (a signed copy is given to the patient).

12.3 Trial insurance

Trial insurance is provided by the Antwerp University Hospital, Drie Eikenstraat 655, 2650 Edegem, BELGIUM.

12.4 Publication policy

The results of this clinical trial will be submitted for publication to an international, peer-reviewed scientific journal. Authors of the manuscript will include at least the Coordinating investigator, Principal investigators Subinvestigators and Study coordinators of this study. All the authors will review the final draft of the manuscript and vouch for its accuracy, completeness and fidelity to the protocol. Acknowledgements to the external project funding listed below will be made in the publication.

13 Ethical considerations

13.1 Patient protection

It is the Investigator's responsibility to ensure that this study is conducted in agreement with either the current revision of the World Medical Association (WMA) Declaration of Helsinki or laws and regulations of the concerned country, whichever provides the greatest protection of the patient. In the Belgian context, the Law of May 7, 2004 ("Wet van 7 mei 2004 inzake experimenten op de menselijke persoon") applies.

In accordance with this law, the study protocol was submitted for approval to the Independent Ethics Committee (IEC). The protocol has been written and the study will be conducted in agreement with the current ICH-GCP guidelines. Any protocol amendments and changes to the informed consent form will need to receive IEC approval before implementation.

Study personnel involved in the execution of this study will be qualified by education, training and experience to perform their respective tasks.

13.2 Informed consent

All patients will be informed of the aims of the study, the possible AEs, the procedures and possible hazards to which the participant will be exposed. They will be informed that participation in the study is voluntary and that they may withdraw from the study at any time and that the withdrawal of consent will not affect her/his subsequent medical treatment or relationship with the treating physician. All patients will be informed as to the strict confidentiality of their patient data, but that their medical records may be reviewed for study purposes by authorized individuals other than their treating physician. All the collected data will be treated in compliance with the European General Data Protection Regulation (GDPR – EU2016/679) and the Belgian legislation that is working on further elaboration of this regulation.

14 Organization of the study

14.1 Trial sponsorship and project funding

Trial sponsorship	Project funding
Antwerp University Hospital (UZA)<br>Drie Eikenstraat 655<br>B-2650 Edegem<br>BELGIUM	Kom op tegen Kanker – Clinical Research Grant – Call for proposals 2021<br><br>NV Roche SA<br>Rue Dante 75<br>1070 Brussels<br>Belgium

All WT1/DC vaccine-related actions (vaccine preparation, vaccine administration, data collection) will be carried out at the Antwerp University Hospital. All other protocol specific actions will be carried out at AZ Middelares or VITAZ.

14.2 Organization of the study - overview

Procedures initiated by Principal Investigator UZA	Shared Procedures	Procedures by Principal Investigators non-UZA
Clinical Trial Coordination<br><br>Screening for leukapheresis (Division of Hematology, UZA and Division of Nephrology, UZA, under the responsibility of the Cell and Tissue Bank, UZA)<br><br>Leukapheresis procedure (Division of Nephrology, UZA, under the responsibility of the Cell and Tissue Bank, UZA)<br><br>GMP DC vaccine manufacturing (anicells)<br><br>DC vaccine preparation (CCRG UZA under responsibility of UZA Pharmacy)<br><br>DC vaccine administration (Division of Hematology, UZA)<br><br>Immunomonitoring (Laboratory of Experimental Hematology, UAntwerp; CCRG and Division of Pathology, UZA)	Possibility of patient inclusion<br><br>Compliance to Study Protocol: Standard of care treatment + atezolizumab with intermittent vaccination<br><br>Deliberation on additional vaccination and/or atezolizumab administration, if applicable<br><br>Patient consent for additional vaccination and/or atezolizumab administration, if applicable<br><br>Patient consent for vaccination after disease progression, if applicable	Patient recruitment<br><br>Patient initial informed consent<br><br>Patient eligibility screening<br><br>Standard of care treatment<br><br>Atezolizumab stock logistics<br><br>Atezolizumab administration<br><br>Tumor evaluation<br><br>Post progressive disease treatment<br><br>Patient follow-up after vaccine and/or atezolizumab treatment discontinuation

14.3 Clinical coordination

14.3.1 Coordinating investigator

Zwi N. Berneman, MD, PhD
Antwerp University Hospital, Division of Hematology
Drie Eikenstraat 655, B-2650 Edegem, BELGIUM
[redacted]

14.3.2 Principal investigators

Subinvestigators are listed in alphabetic order.

Zwi N. Berneman, MD, PhD
Antwerp University Hospital, Division of Hematology
Drie Eikenstraat 655, B-2650 Edegem, BELGIUM
[redacted]

Koen Deschepper, MD
VITAZ, Division of Pulmonary and Infectious Diseases
Moerlandstraat 1, B-9100 Sint-Niklaas, BELGIUM
[redacted]

Paul Germonpré, MD, PhD
AZ Middelares, Respiratory Oncology & Integrated Cancer Center Ghent
Buitenring St.-Denijs 30, B-9000 Ghent, BELGIUM
[redacted]

14.3.3 Subinvestigator

[redacted]
Antwerp University Hospital, Division of Hematology
University of Antwerp (UAntwerp)
Drie Eikenstraat 655, B-2650 Edegem, BELGIUM
[redacted]

14.3.4 Study coordinators

Study coordinators are listed in alphabetic order.

[redacted]
Antwerp University Hospital, CCRG
Drie Eikenstraat 655, B-2650 Edegem, BELGIUM
[redacted]

[redacted]
Antwerp University Hospital, CCRG
University of Antwerp, Laboratory of Experimental Hematology (LEH)
Drie Eikenstraat 655, B-2650 Edegem, BELGIUM
[redacted]

[redacted]
Antwerp University Hospital, CCRG
University of Antwerp, LEH
Drie Eikenstraat 655, B-2650 Edegem, BELGIUM
[redacted]

14.4 Patient recruitment

Patients will be recruited at the

Respiratory Oncology & Integrated Cancer Center Ghent
AZ Maria Middelares
Paul Germonpré, MD, PhD
Buitenring St.-Denijs 30, B-9000 Ghent, BELGIUM
[redacted]

Division of Pulmonary and Infectious Diseases
VITAZ
Koen Deschepper, MD
Moerlandstraat 1, B-9100 Sint-Niklaas, BELGIUM
[redacted]

The study will also be open to other referring centers.

14.5 Data management

Data managers will be responsible for:

- ensuring compliance with the clinical trial protocol
- reviewing patient eligibility criteria
- registering eligible patients as per the study protocol
- collecting and reviewing electronic Case Report Forms (eCRFs)

Center for Cell Therapy and Regenerative Medicine (CCRG)
Antwerp University Hospital (UZA)
Drie Eikenstraat 655, B-2650 Edegem, BELGIUM
[redacted]

Division of Pneumology-Oncology
AZ Maria Middelares
Buitenring St.-Denijs 30, B-9000 Ghent, BELGIUM
[redacted]

Division of Pulmonary and Infectious Diseases
VITAZ
Moerlandstraat 1, B-9100 Sint-Niklaas, BELGIUM

[redacted]

14.6 Leukapheresis unit

Leukapheresis will be performed at the Division of Nephrology-Hypertension of UZA, under responsibility of the Cell and Tissue Bank UZA .

Division of Nephrology-Hypertension
Antwerp University Hospital (UZA)
Drie Eikenstraat 655, B-2650 Edegem, BELGIUM
[redacted]

14.7 Vaccine manufacturing

Cryopreserved WT1/DC vaccines will be manufactured according to the latest version of the IMPD in compliance with GMP standards and all other applicable regulations and requirements, including those prescribed by the Belgian Federal Agency for Medicines and Health Products (FAGG/AFMPS).

Manufacturer: anicells
Galileilaan 21, B-2845 Niel, BELGIUM
[redacted]

14.8 Vaccine preparation site

WT1/DC vaccine preparation for administration according to the latest version of the reconstitution handling manual will be performed at the UZA CCRG facility under responsibility of the hospital pharmacy as agreed in the service level agreement.

Pharmacy UZA
Antwerp University Hospital (UZA)
Drie Eikenstraat 655, B-2650 Edegem, BELGIUM
[redacted]

Center for Cell Therapy and Regenerative Medicine (CCRG)
Antwerp University Hospital (UZA)
Drie Eikenstraat 655, B-2650 Edegem, BELGIUM
[redacted]

14.9 Vaccination site

WT1/DC vaccines will be administered by the Division of Hematology of the Antwerp University Hospital.

Division of Hematology
Antwerp University Hospital (UZA)
Drie Eikenstraat 655, B-2650 Edegem - BELGIUM
[redacted]

14.10 Platinum/pemetrexed-based chemotherapy and atezolizumab administration

Platinum/pemetrexed-based chemotherapy and atezolizumab will be administered under authorization of the Investigators in the below mentioned specialized experienced oncology centers .

Respiratory Oncology & Integrated Cancer Center Ghent
AZ Middelares
[redacted]
Buitenring St.-Denijs 30, B-9000 Ghent, BELGIUM
[redacted]

Division of Pulmonary and Infectious Diseases
VITAZ
[redacted]
Moerlandstraat 1, B-9100 Sint-Niklaas, BELGIUM
[redacted]

14.11 Immunomonitoring sites

Immunomonitoring studies on blood samples and tumor biopsies (if available) will be performed at CCRG of the Antwerp University Hospital and the Laboratory of Experimental Hematology (LEH) of the University of Antwerp in collaboration with other divisions, including but not restricted to, the division of Pathology of the Antwerp University Hospital, Advanced Database Research and Modelling (ADReM) group of the University of Antwerp, the Biomedical Informatics Research Center Antwerp (biomina) at the University of Antwerp and the Antwerp University Hospital and the Center for Medical Genetics, as detailed in the respective internal service level agreements.

Center for Cell Therapy and Regenerative Medicine (CCRG)
Antwerp University Hospital (UZA)
Drie Eikenstraat 655, B-2650 Edegem, BELGIUM
[redacted]

Laboratory of Experimental Hematology (LEH)
University of Antwerp (UAntwerp)
Drie Eikenstraat 655, B-2650 Edegem, BELGIUM
[redacted]

14.12 Data monitoring

Data monitoring visits will be performed by the Clinical Trial Center (CTC) of the Antwerp University Hospital.

Clinical Trial Center Antwerpen
Antwerp University Hospital (UZA)
Drie Eikenstraat 655, B-2650 Edegem, BELGIUM
[redacted]

14.13 Statistical analyses

Statistical analyses will be performed by statisticians from the Clinical Trial Center of the Antwerp University Hospital.

Clinical Trial Center Antwerpen
Antwerp University Hospital (UZA)
Drie Eikenstraat 655, B-2650 Edegem, BELGIUM
[redacted]

15 References

1. Robinson BW, Musk AW, Lake RA. Malignant mesothelioma. *Lancet* 2005; **366**(9483): 397-408.

2. Robinson BW, Lake RA. Advances in malignant mesothelioma. *N Engl J Med* 2005; **353**(15): 1591-603.

3. Alpert N, van Gerwen M, Taioli E. Epidemiology of mesothelioma in the 21(st) century in Europe and the United States, 40 years after restricted/banned asbestos use. *Translational lung cancer research* 2020; **9**(Suppl 1): S28-s38.

4. Vogelzang NJ, Rusthoven JJ, Symanowski J, et al. Phase III study of pemetrexed in combination with cisplatin versus cisplatin alone in patients with malignant pleural mesothelioma. *J Clin Oncol* 2003; **21**(14): 2636-44.

5. Musk AW, Olsen N, Alfonso H, et al. Predicting survival in malignant mesothelioma. *Eur Respir J* 2011; **38**(6): 1420-4.

6. Scherpereel A, Wallyn F, Albelda SM, Munck C. Novel therapies for malignant pleural mesothelioma. *Lancet Oncol* 2018; **19**(3): e161-e72.

7. Bagia M, Nowak AK. Novel targeted therapies and vaccination strategies for mesothelioma. *Curr Treat Options Oncol* 2011; **12**(2): 149-62.

8. Baas P, Scherpereel A, Nowak AK, et al. First-line nivolumab plus ipilimumab in unresectable malignant pleural mesothelioma (CheckMate 743): a multicentre, randomised, open-label, phase 3 trial. *Lancet* 2021; **397**(10272): 375-86.

9. Banchereau J, Steinman RM. Dendritic cells and the control of immunity. *Nature* 1998; **392**(6673): 245-52.

10. Palucka K, Banchereau J. Cancer immunotherapy via dendritic cells. *Nat Rev Cancer* 2012; **12**(4): 265-77.

11. Sprooten J, Ceusters J, Coosemans A, et al. Trial watch: dendritic cell vaccination for cancer immunotherapy. *Oncoimmunology* 2019; **8**(11): e1638212.

12. Hsu FJ, Benike C, Fagnoni F, et al. Vaccination of patients with B-cell lymphoma using autologous antigen-pulsed dendritic cells. *Nat Med* 1996; **2**(1): 52-8.

13. Garg AD, Vara Perez M, Schaaf M, et al. Trial watch: Dendritic cell-based anticancer immunotherapy. *Oncoimmunology* 2017; **6**(7): e1328341.

14. Van Tendeloo VF, Van de Velde A, Van Driessche A, et al. Induction of complete and molecular remissions in acute myeloid leukemia by Wilms' tumor 1 antigen-targeted dendritic cell vaccination. *Proc Natl Acad Sci U S A* 2010; **107**(31): 13824-9.

15. Anguille S, Van de Velde AL, Smits EL, et al. Dendritic cell vaccination as postremission treatment to prevent or delay relapse in acute myeloid leukemia. *Blood* 2017; **130**(15): 1713-21.

16. Berneman ZN, Anguille S, Willemen Y, et al. Vaccination of cancer patients with dendritic cells electroporated with mRNA encoding the wilms' tumor 1 protein (WT1): correlation of clinical effect and overall survival with T-cell response. *Cytotherapy* 2019; **21**(5): S10.

17. Izzi V, Masuelli L, Tresoldi I, Foti C, Modesti A, Bei R. Immunity and malignant mesothelioma: From mesothelial cell damage to tumor development and immune response-based therapies. *Cancer Lett* 2012.

18. Gregoire M. What's the place of immunotherapy in malignant mesothelioma treatments? *Cell Adh Migr* 2010; **4**(1): 153-61.

19. Kushitani K, Takeshima Y, Amatya VJ, Furonaka O, Sakatani A, Inai K. Immunohistochemical marker panels for distinguishing between epithelioid mesothelioma and lung adenocarcinoma. *Pathol Int* 2007; **57**(4): 190-9.

20. Van Tendeloo VF, Ponsaerts P, Lardon F, et al. Highly efficient gene delivery by mRNA electroporation in human hematopoietic cells: superiority to lipofection and passive pulsing of mRNA and to electroporation of plasmid cDNA for tumor antigen loading of dendritic cells. *Blood* 2001; **98**(1): 49-56.

21. Smits EL, Anguille S, Cools N, Berneman ZN, Van Tendeloo VF. Dendritic cell-based cancer gene therapy. *Hum Gene Ther* 2009; **20**(10): 1106-18.

22. Benteyn D, Anguille S, Van Lint S, et al. Design of an Optimized Wilms' Tumor 1 (WT1) mRNA Construct for Enhanced WT1 Expression and Improved Immunogenicity In Vitro and In Vivo. *Molecular therapy Nucleic acids* 2013; **2**: e134.

23. Hillerdal G, Sorensen JB, Sundström S, Riska H, Vikström A, Hjerpe A. Treatment of malignant pleural mesothelioma with carboplatin, liposomized doxorubicin, and gemcitabine: a phase II study. *J Thorac Oncol* 2008; **3**(11): 1325-31.

24. Scherpereel A, Mazieres J, Greillier L, et al. Nivolumab or nivolumab plus ipilimumab in patients with relapsed malignant pleural mesothelioma (IFCT-1501 MAPS2): a multicentre, open-label, randomised, non-comparative, phase 2 trial. *Lancet Oncol* 2019; **20**(2): 239-53.

25. Quispel-Janssen J, van der Noort V, de Vries JF, et al. Programmed Death 1 Blockade With Nivolumab in Patients With Recurrent Malignant Pleural Mesothelioma. *J Thorac Oncol* 2018; **13**(10): 1569-76.

26. Fennell D OC, Califano R. Nivolumab versus placebo in relapsed malignant mesothelioma: The CONFIRM phase 3 trial. 2020 World Conference on Lung Cancer; 2021; WCLC 2020 Virtual; 2021.

27. Fennell DA, Kirkpatrick E, Cozens K, et al. CONFIRM: a double-blind, placebo-controlled phase III clinical trial investigating the effect of nivolumab in patients with relapsed mesothelioma: study protocol for a randomised controlled trial. *Trials* 2018; **19**(1): 233.

28. Disselhorst MJ, Quispel-Janssen J, Lalezari F, et al. Ipilimumab and nivolumab in the treatment of recurrent malignant pleural mesothelioma (INITIATE): results of a prospective, single-arm, phase 2 trial. *Lancet Respir Med* 2019; **7**(3): 260-70.

29. Garg AD, Coulie PG, Van den Eynde BJ, Agostinis P. Integrating Next-Generation Dendritic Cell Vaccines into the Current Cancer Immunotherapy Landscape. *Trends Immunol* 2017; **38**(8): 577-93.

30. van Gulijk M, Dammeijer F, Aerts J, Vroman H. Combination Strategies to Optimize Efficacy of Dendritic Cell-Based Immunotherapy. *Front Immunol* 2018; **9**(2759): 2759.

31. Oh SA, Wu D-C, Cheung J, et al. PD-L1 expression by dendritic cells is a key regulator of T-cell immunity in cancer. *Nature Cancer* 2020.

32. Park SJ, Namkoong H, Doh J, et al. Negative role of inducible PD-1 on survival of activated dendritic cells. *J Leukoc Biol* 2014; **95**(4): 621-9.

33. Versteven M, Van den Bergh JMJ, Marcq E, et al. Dendritic Cells and Programmed Death-1 Blockade: A Joint Venture to Combat Cancer. *Front Immunol* 2018; **9**: 394.

34. Van den Bergh JMJ, Smits E, Berneman ZN, et al. Monocyte-Derived Dendritic Cells with Silenced PD-1 Ligands and Transpresenting Interleukin-15 Stimulate Strong Tumor-Reactive T-cell Expansion. *Cancer Immunol Res* 2017; **5**(8): 710-5.

35. Zaidi N, Quezada SA, Kuroiwa JMY, et al. Anti-CTLA-4 synergizes with dendritic cell-targeted vaccine to promote IL-3-dependent CD4(+) effector T cell infiltration into murine pancreatic tumors. *Ann N Y Acad Sci* 2019; **1445**(1): 62-73.

36. Nagaoka K, Hosoi A, Iino T, Morishita Y, Matsushita H, Kakimi K. Dendritic cell vaccine induces antigen-specific CD8(+) T cells that are metabolically distinct from those of peptide vaccine and is well-combined with PD-1 checkpoint blockade. *Oncoimmunology* 2018; **7**(3): e1395124.

37. Ge Y, Xi H, Ju S, Zhang X. Blockade of PD-1/PD-L1 immune checkpoint during DC vaccination induces potent protective immunity against breast cancer in hu-SCID mice. *Cancer Lett* 2013; **336**(2): 253-9.

38. Broos K, Lecocq Q, Keersmaecker B, et al. Single Domain Antibody-Mediated Blockade of Programmed Death-Ligand 1 on Dendritic Cells Enhances CD8 T-cell Activation and Cytokine Production. *Vaccines (Basel)* 2019; **7**(3): 85.

39. Versteven M, Van den Bergh JMJ, Broos K, et al. A versatile T cell-based assay to assess therapeutic antigen-specific PD-1-targeted approaches. *Oncotarget* 2018; **9**(45): 27797-808.

40. Emens LA, Middleton G. The interplay of immunotherapy and chemotherapy: harnessing potential synergies. *Cancer Immunol Res* 2015; **3**(5): 436-43.

41. Antonia SJ, Mirza N, Fricke I, et al. Combination of p53 cancer vaccine with chemotherapy in patients with extensive stage small cell lung cancer. *Clin Cancer Res* 2006; **12**(3 Pt 1): 878-87.

42. Wheeler CJ, Das A, Liu G, Yu JS, Black KL. Clinical responsiveness of glioblastoma multiforme to chemotherapy after vaccination. *Clin Cancer Res* 2004; **10**(16): 5316-26.

43. Fehrenbacher L, Spira A, Ballinger M, et al. Atezolizumab versus docetaxel for patients with previously treated non-small-cell lung cancer (POPLAR): a multicentre, open-label, phase 2 randomised controlled trial. *Lancet* 2016; **387**(10030): 1837-46.

44. Rosenberg JE, Hoffman-Censits J, Powles T, et al. Atezolizumab in patients with locally advanced and metastatic urothelial carcinoma who have progressed following treatment with platinum-based chemotherapy: a single-arm, multicentre, phase 2 trial. *Lancet* 2016; **387**(10031): 1909-20.

45. Van Driessche A, Berneman ZN, Van Tendeloo VF. Active specific immunotherapy targeting the Wilms' tumor protein 1 (WT1) for patients with hematological malignancies and solid tumors: lessons from early clinical trials. *Oncologist* 2012; **17**(2): 250-9.

46. Anguille S, Smits EL, Lion E, Van Tendeloo VF, Berneman ZN. Clinical use of dendritic cells for cancer therapy. *The Lancet Oncology* 2014.

16 Appendices

16.1 Appendix A: Laboratory tests

TEST		TIMING
**Hematology**	erythrocytes/hematocrit, hemoglobin, thrombocytes, leukocytes, leukocyte formula, reticulocytes	screening visits *(all centers)*<br>leukapheresis (*UZA)*<br>patient evaluation time points *(Section 5.2.4.1)*
**Biochemistry**	creatinine, urea*, sodium, potassium, chloride, bicarbonate, calcium, phosphor, uric acid, CRP, total protein, glucose, LDH, AST, ALT, ALP, ©-GT, bilirubin (including fractions), magnesium, lipase, ferritin, iron + TIBC, albumin**, amylase, CK, troponin, TSH	screening visits *(all centers)*<br>leukapheresis *(UZA)*<br>patient evaluation time points *(Section 5.2.4.1)*
**Biochemistry**	protein electrophoresis<br>folic acid	screening visits *(all centers)*
**Serology**	HIV1 Antibody/Antigen, HIV2 Antibody/Antigen, HepB sAntigen, HepB sAntibody, HepB cAntibody, HepC Antibody	screening visit *(VITAZ and AZMM)*<br>leukapheresis *(UZA)*
**Serology**	Syphilis RPR, Syphilis TPHA	screening visit (UZA)<br>leukapheresis (UZA)
**Hemostasis**	APTT, PT	screening visit *(all centers)*<br>leukapheresis *(UZA)*
	fibrinogen, D-dimer	screening visit (UZA)<br>leukapheresis (UZA)
**Blood group**	blood group ABO	screening visit *(UZA)*<br>leukapheresis *(UZA)*
**Rhesus factor**	rhesus factor	screening visit *(UZA)*<br>leukapheresis *(UZA)*
**Pregnancy** *(if applicable)*	HCG	screening visit *(all centers)*

* Testing for urea will only be performed when glomerular filtration <30mL/min/1,73m^2 (value derived from creatinine test)
** Albumin at screening will be derived from the test for protein electrophoresis.
